# Replication of synthetic recognition-encoded oligomers by ligation of trimer building blocks†

**DOI:** 10.1039/d3qo01717f

**Published:** 2023-10-25

**Authors:** Diego Núñez-Villanueva, Christopher A. Hunter

**Affiliations:** a Yusuf Hamied Department of Chemistry, University of Cambridge Lensfield Road Cambridge CB2 1EW UK herchelsmith.orgchem@ch.cam.ac.uk

## Abstract

The development of methods for replication of synthetic information oligomers will underpin the use of directed evolution to search new chemical space. Template-directed replication of triazole oligomers has been achieved using a covalent primer in conjunction with non-covalent binding of complementary building blocks. A phenol primer equipped with an alkyne was first attached to a benzoic recognition unit on a mixed sequence template *via* selective covalent ester base-pair formation. The remaining phenol recognition units on the template were then used for non-covalent binding of phosphine oxide oligomers equipped with an azide. The efficiency of the templated CuAAC reaction between the primer and phosphine oxide building blocks was investigated as a function of the number of H-bonds formed with the template. Increasing the strength of the non-covalent interaction between the template and the azide lead to a significant acceleration of the templated reaction. For shorter phosphine oxide oligomers intermolecular reactions compete with the templated process, but quantitative templated primer elongation was achieved with a phosphine oxide 3-mer building block that was able to form three H-bonds with the template. NMR spectroscopy and molecular models suggest that the template can fold, but addition of the phosphine oxide 3-mer leads to a complex with three H-bonds between phosphine oxide and phenol groups, aligning the azide and alkyne groups in a favourable geometry for the CuAAC reaction. In the product duplex, ^1^H and ^31^P NMR data confirm the presence of the three H-bonded base-pairs, demonstrating that the covalent and non-covalent base-pairs are geometrically compatible. A complete replication cycle was carried out starting from the oligotriazole template by covalent attachment of the primer, followed by template-directed elongation, and hydrolysis of the the ester base-pair in the resulting duplex to regenerate the template and liberate the copy strand. We have previously demonstrated sequence-selective oligomer replication using covalent base-pairing, but the trimer building block approach described here is suitable for replication of sequence information using non-covalent binding of the monomer building blocks to a template.

## Abstract 2

The use of a building block that make three non-covalent base-pairs with a template oligomer leads to high affinity binding and high fidelity replication in a coupling reaction with a primer attached to the template with a covalent base-pair.

## Graphical abstract

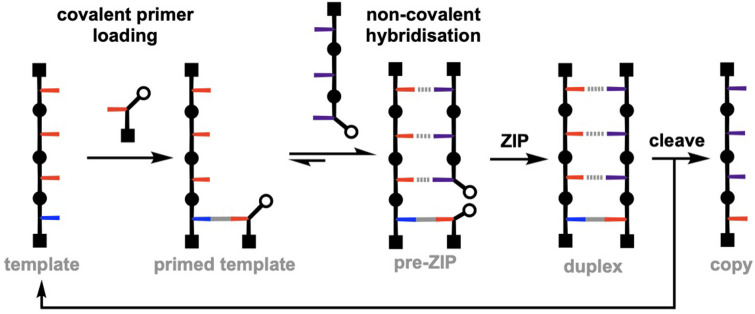

## Introduction

Replication of chemical information is the molecular foundation for the evolution of living organisms.^1^ Sequence information transfer takes place *via* template-directed synthesis from nucleic acid templates. This process, optimised by millions of years of evolution, has been exploited in the development of breakthrough techniques, namely PCR and SELEX.^2–6^ Directed evolution is a powerful method for tailoring biomolecules and has led to advances in the search for novel therapeutic agents and more efficient and sustainable manufacturing processes.^7–9^ To date, the transfer of information from a parent template to a daughter oligomer in such techniques relies exclusively on nucleic acids.^10–12^ However, there is potential to extend molecular evolution techniques to synthetic systems and access new regions of unexplored chemical space.^13,14^ The physicochemical properties of such synthetic oligomers will depend on the chemical structure of the backbone and the functional groups that encode information, allowing exploitation in different environments from nucleic acids, *i.e.* in organic solvents, low temperature, *etc*.^15–17^ The functional properties of these synthetic oligomers would be defined by the sequence of the monomer units in the chain, which could be optimised by evolution.^18–20^ A key requirement for the use of synthetic oligomers in molecular evolution is an efficient method to copy the encoded sequence information into a daughter strand.

**Fig. 1:**
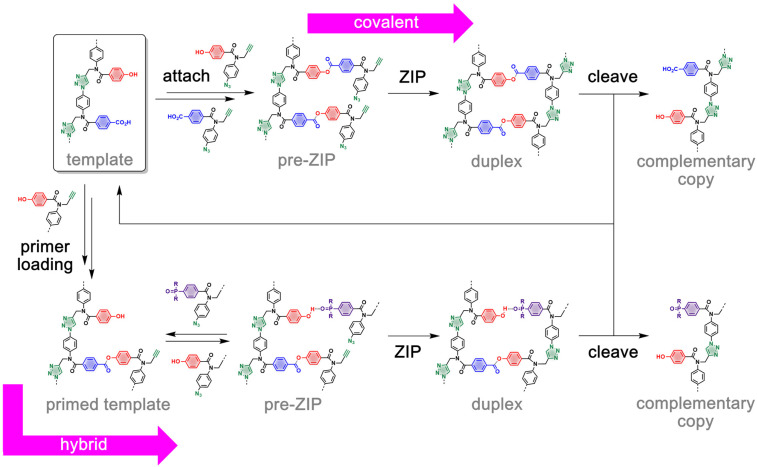
Two strategies for the transfer of sequence information encoded in triazole oligomers: fully covalent and hybrid covalent/non-covalent replication. The top pathway shows a covalent template-directed replication method. In the first step, phenol and benzoic acid monomers are orthogonally attached to the complementary base by ester coupling. Intramolecular CuAAC oligomerization of the pre-ZIP intermediate yields the covalent duplex, which can be cleaved to release the template along with the complementary copy. In the bottom pathway, both covalent and non-covalent base-pairs are simultaneously used in primer-assisted replication. In the first step, a covalent primer is orthogonally loaded onto the template by ester coupling. Then, H-bond interactions between phenol units of the primed template and complementary phosphine oxide monomers lead to the formation of a hybrid pre-ZIP complex. Intramolecular CuAAC reaction provides the corresponding duplex, and cleavage of the covalent ester base-pair releases the template and the complementary copy.

We recently described the use of covalent templating to achieve efficient sequence information transfer between synthetic triazole oligomers (Fig. 1, top channel).^21,22^ The base-pairing system involves kinetically inert ester bonds between phenol and benzoic acid side-chains, and copper catalyzed azide alkyne cycloaddition (CuAAC) is used for oligomerization of the backbone of the daughter strand. The resulting covalent duplex can be cleaved by ester hydrolysis to regenerate the initial template along with the complementary copy. The use of covalent base-pairs overcomes the issue of incomplete loading of monomers onto the template when weaker non-covalent interactions are used. It also enables reprogramming of the information transfer process *via* the use of traceless linkers to attach monomers to the template, so that direct replication, reciprocal replication and mutation are all accessible.^23–25^

We have extended the use of covalent base-pairs to develop an alternative replication method, inspired by the polymerase chain reaction (PCR) of nucleic acids. A key element in PCR is the use of primers, which define the region of the single-stranded template to be elongated. In non-enzymatic nucleic acid replication, a primer is also essential for template-directed synthesis of the complementary strand. The primer must have a high affinity for the template to avoid non-templated ligation reactions, which requires the design of long primers for effective annealing or the use of excess template to ensure that the primer is fully template bound.^26,27^

With the aim of developing efficient replication methods for synthetic oligomers, we recently described a hybrid template-directed primer extension method, combining covalent and non-covalent base-pairing (Fig. 1, bottom channel).^28^ A cleavable covalent ester base-pair was used for efficient primer loading, *via* a benzoic acid unit at one end of the template. This kinetically stable attachment enables the use of short and simple primers, in contrast to nucleic acid replication. The resulting primed template has phenol H-bond donors that form strong non-covalent interactions with phosphine oxide H-bond acceptor monomers. The primer is equipped with an alkyne for CuAAC elongation with azide monomers. Phenol-phosphine oxide H-bonding interactions lead to the pre-ZIP complex, which templates the CuAAC reaction of the monomer with the primer. Hydrolysis of the template-primer ester bond in the resulting duplex releases the complementary copy from the template.

**Fig. 2:**
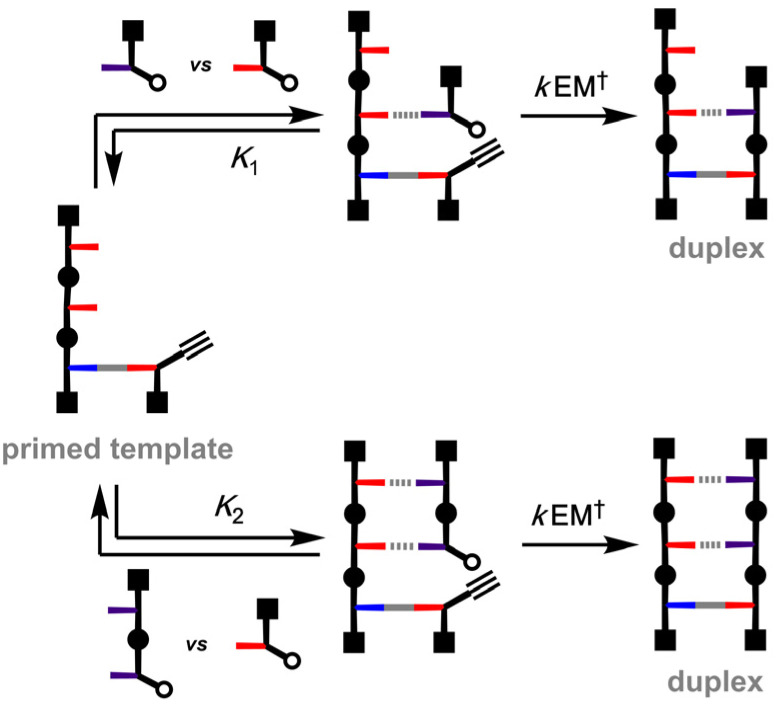
Schematic representation of the competition reaction between phenol (red) and phosphine oxide (purple) azides for the primer alkyne loaded onto the template. *K*_1_ and *K*_2_ are the binding constants of phosphine oxide 1-mer and 2-mer with the template, respectively. *k* is the intrinsic rate constant for the CuAAC reaction in non-templating conditions while EM† is the kinetic effective molarity for the templated reaction.

Fig. 2 shows the key parameters determining the efficiency of the non-covalent template-directed primer elongation when a 3-mer template is used with different types of azide monomer.^28^ The template has one benzoic acid unit (blue), used for covalent attachment of the primer, and two phenol groups (red) for H-bonding with phosphine oxides (purple). When a phosphine oxide 1-mer is used (Fig. 2, top channel), the first step involves binding to the template (*K*_1_ ≈ 200 M^−1^ in dichloromethane). The rate of the templated CuAAC reaction is given by the product of the rate constant for the untemplated reaction (*k*) and the kinetic effective molarity for the intramolecular process (EM^†^). The rate constant for a competing untemplated reaction with a phenol 1-mer is *k*. The success of the templating process is therefore related to the product of *K*_1_ and EM^†^. The value of EM^†^ depends on the geometric compatibility of the template-monomer complex with the transition state for the CuAAC reaction and on backbone flexibility and is therefore rather difficult to predict or control. In contrast, the binding affinity of the azide monomer (*K*_1_) for the template can be manipulated in a straightforward manner.

We have shown previously that the use of a phosphine oxide 2-mer leads to a significant acceleration of the templated reaction (Fig. 2 bottom channel), because *K*_2_ is one order of magnitude higher than *K*_1_ (*K*_2_ ≈ 1500 M^−1^ in dichloromethane). Similar phenomena have been described in non-enzymatic nucleic acid replication, where the use of activated oligomers instead of monomers significantly increases the efficiency of primer extension.^29^ Here we show how this approach can be developed further to achieve near quantitative template-directed primer extension of an oligotriazole.

## Results and discussion

The oligotriazole architecture highlighted in Fig. 1 is ideal for further optimization of the templating process, because the binding affinity between two oligomers increases by one order of magnitude for each H-bonded base-pair added to the chain. Increasing the length of the monomer building blocks therefore provides a convenient tool for improving the efficiency of the templated CuAAC reaction by increasing the stability of the pre-ZIP complex. We report here the output of the templating process for a 4-mer template and an azide-functionalised phosphine oxide 3-mer used to elongate the covalent primer.

**Scheme 1:**
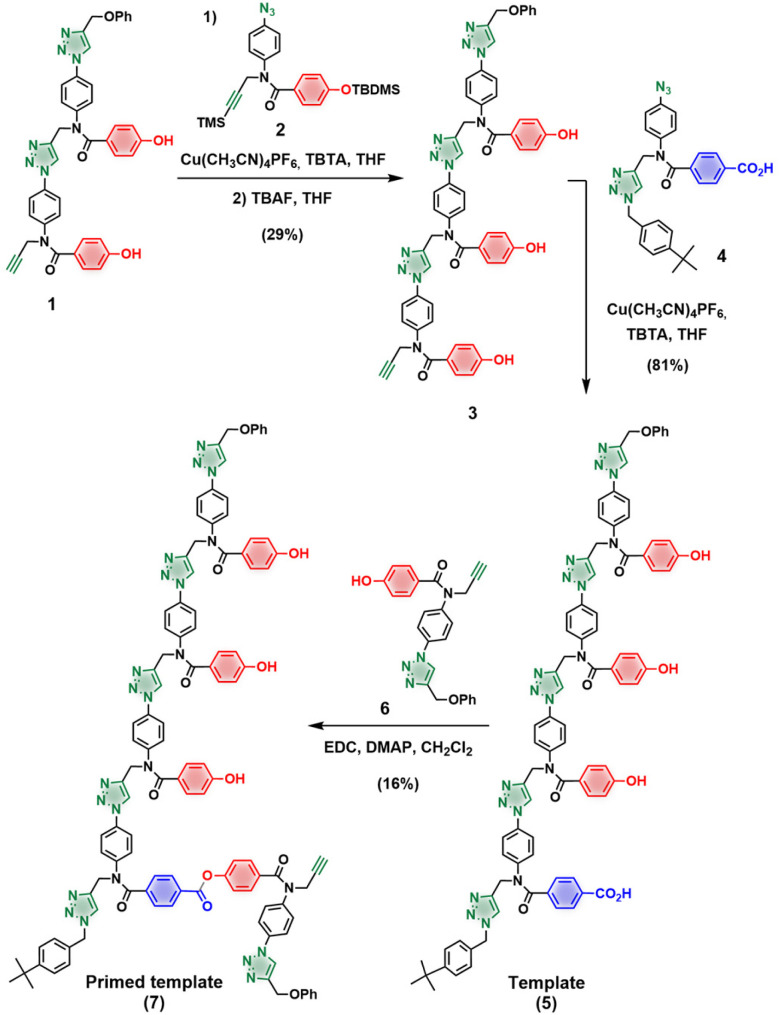
Synthesis of template 5 and primed template 7.

**Scheme 2:**
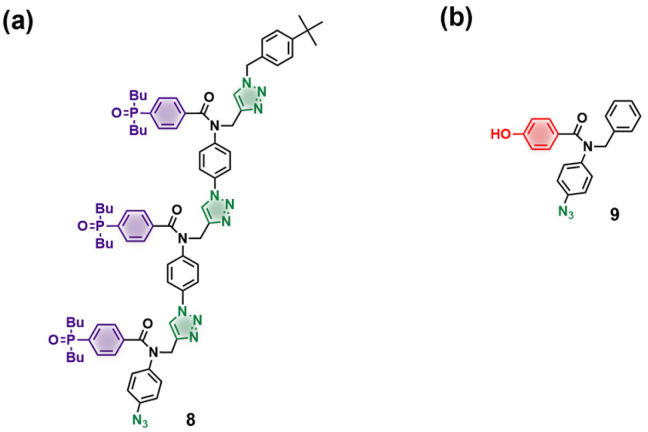
(a) Phosphine oxide 3-mer azide, prepared by oligomerization of a phosphine oxide 1-mer bearing an alkyne and an azide in the presence of a 4-*tert*-butylbenzyl azide end-capping group. (b) Phenol 1-mer azide.^28^

Synthesis of the 4-mer template containing three H-bonding phenol units and the subsequent incorporation of the covalent primer is shown in Scheme 1. CuAAC coupling between the phenol 2-mer 1 ^21^ and the protected azide 2,^21^ followed by TBAF-mediated removal of the silyl groups afforded phenol 3-mer 3. The terminal alkyne in 3 was reacted with capped benzoic acid 1-mer 4 to yield template 5 in good yield. The primer was loaded onto the template by ester coupling with an excess of phenol 1-mer 6, affording primed template 7. Scheme 2 shows the 3-mer phosphine oxide azide 8 and the phenol azide 9 used to assess the template effect in competition reactions.^28^

**Fig. 3:**
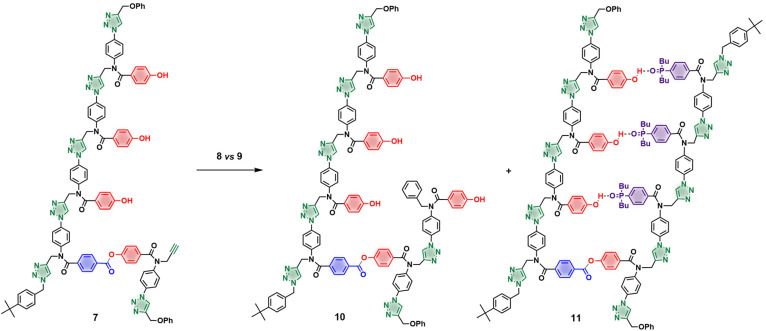
Competition CuAAC reaction used to quantify template effects.

**Fig. 4:**
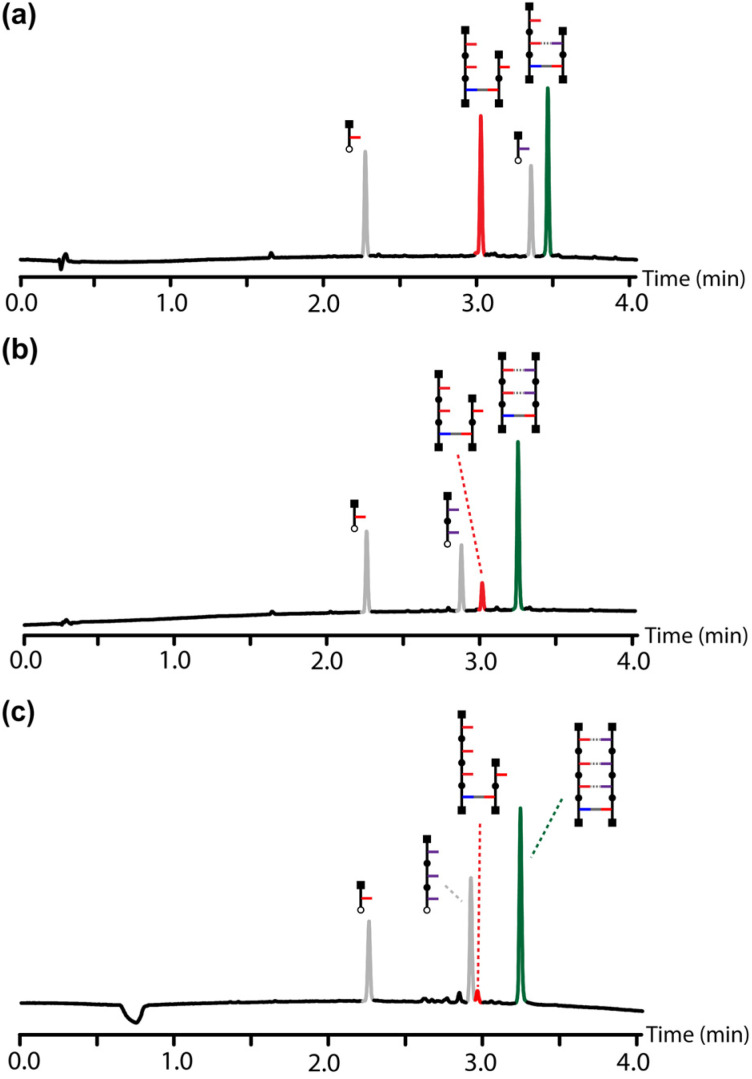
UPLC traces of crude reaction mixtures from competition experiments using equimolar mixtures of two azides: (a) phosphine oxide 1-mer and phenol 9; (b) phosphine oxide 2-mer and phenol 9; (c) phosphine oxide 3-mer and phenol 9. Reaction conditions: [primed template] = 0.1 mM, [phosphine oxide azide] = 0.15 mM, [phenol azide] = 0.15 mM, [Cu(CH_3_CN)_4_PF_6_·TBTA] = 0.2 mM in CH_2_Cl_2_, stirring at room temperature for 48 h. UPLC conditions: C18 column at 40 °C (270 nm) using water + 0.1% formic acid (A) and CH_3_CN + 0.1% formic acid (B); gradient of 0–4 min 5%–100% B + 1 min 100% B.

The primed template 7 was used in a competition reaction between phosphine oxide 3-mer 8 and phenol 9 to assess the template effect (Fig. 3). All reactions were performed in dry and degassed dichloromethane using Cu(CH_3_CN)_4_PF_6_, TBTA and an equimolar excess of each azide. Fig. 4c shows the UPLC trace of the crude reaction mixture after 2 days. The templated product (11, green peak) is formed almost exclusively, with only traces of untemplated product (12, red peak). As a control, the same reaction was performed with a simple alkyne instead of the primed template in order to quantify any difference in the intrinsic reactivity of the two azides (see ESI† for details). In this case, 9 was found to be twice as reactive as 8, which means that the efficiency of the templating process is even higher than Fig. 4c suggests.

Fig. 4a and b show the corresponding results for template-directed primer elongation with a phosphine oxide 1-mer and 2-mer respectively. When the azide makes one H-bond with the template, there are no significant differences between the amounts of the templated (green) and untemplated (red) products (Fig. 4a). When the azide makes two H-bonds with the template, there is a significant acceleration in the rate of the templated pathway and an increase in the yield of the templated product (green) relative to the untemplated product (red). However, substantial amounts of the untemplated product are formed in Fig. 4b. It is only with three H-bonds that the off-template pathway is almost completely supressed, and the template-directed primer elongation product is formed efficiently (Fig. 4c).

**Fig. 5:**
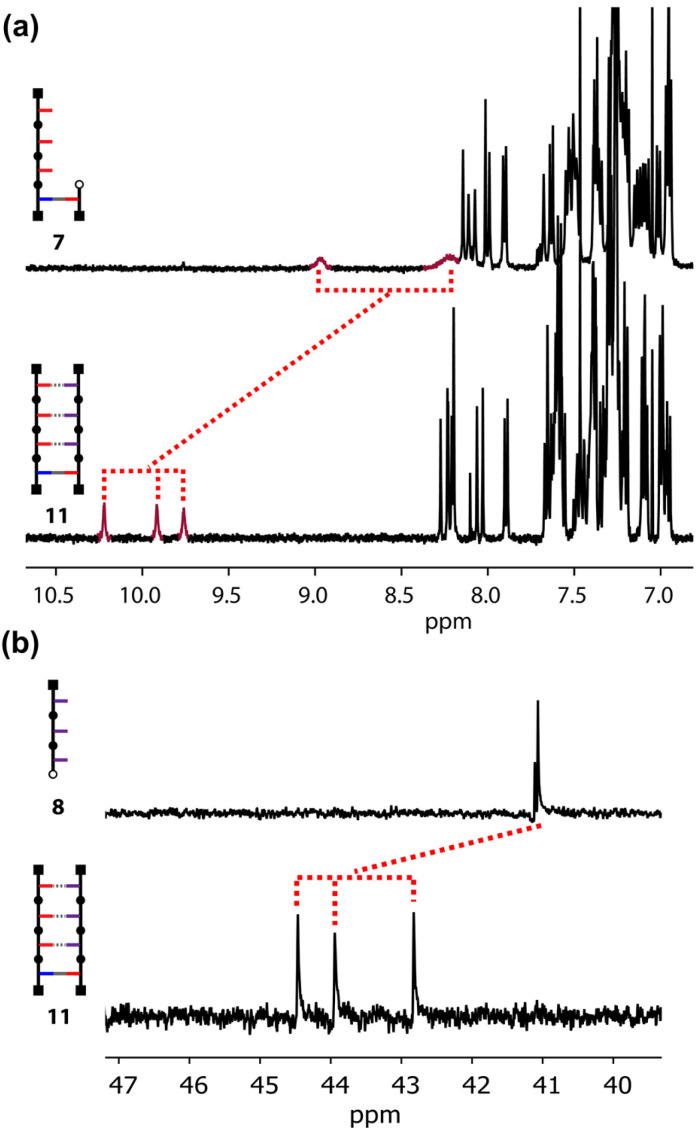
(a) ^1^H NMR spectra (500 MHz) recorded in CDCl_3_ at 298 K of primed template 7 (0.7 mM, top) and duplex 11 (0.4 mM, bottom). The dotted lines connect the signals due to the OH groups. (b) ^31^P NMR spectra (202 MHz) recorded in CDCl_3_ at 298 K of phosphine oxide 3-mer 8 (0.6 mM, top) and duplex 11 (0.4 mM, bottom).

Evidence for the H-bonding interactions involved in templating the CuAAC reaction between the primed template 7 and 3-mer 8 can be obtained from NMR spectra. Fig. 5a shows the ^1^H NMR spectra of primed template 7 and duplex 11 in CDCl_3_. The signals corresponding to the OH groups of 7 appear at 8–9 ppm, which suggest that phenols are involved in H-bonds.^28^ There is no self-association at these concentrations, so there is probably some folding of the primed template due to intramolecular H-bonding between the phenol groups and the carbonyl H-bond acceptors on the backbone. In the duplex 11, the three OH signals are clearly resolved and appear as sharp singlets at 10 ppm, which indicates that they are fully H-bonded to the three phosphine oxide acceptors on the copy strand. Additional evidence for these base-pairing interactions was obtained from ^31^P NMR spectra (Fig. 5b). In duplex 11, the three signals due to the phosphine oxide groups show downfield shifts of 2–4 ppm compared with the 3-mer phosphine oxide 8 where no H-bonding is possible. Thus, the NMR data suggest that the H-bonding interactions that lead to acceleration of the rate of the templated reaction live on in the product, demonstrating that the covalent and non-covalent base-pairs can be simultaneously accommodated within the duplex structure.

**Fig. 6:**
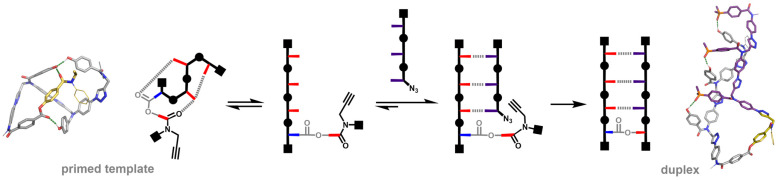
Conformational equilibria involved in assembly of the pre-ZIP complex between 7 and 8. The primed template 7 is partially folded. Complexation with the complementary phosphine oxide 3-mer 8 opens up the template to give the pre-ZIP complex. CuAAC reaction leads to irreversible formation of the duplex 11. Lowest energy structures from conformational searches using molecular mechanics are shown for the primed template and the duplex (MMFFs force field with chloroform solvation with H-bonds as green dotted lines).^30,31^

The NMR data suggest that the templated pathway may be affected by competition with folding of the primed template. Molecular mechanics calculations were performed to obtain further insight into the conformational properties of the template and the duplex (Fig. 6).^30,31^ For the primed template 7, the lowest energy conformation in chloroform indicates that the phenol groups are able to form intramolecular H-bonds with the carbonyl groups of the covalent primer.^30,31^ This structure is consistent with the ^1^H NMR spectrum of 7, which indicates partially H-bonded phenol groups.

The lowest energy structure for the duplex in chloroform is an extended conformation with three phenol-phosphine oxide H-bonds, which is again consistent with the NMR data. The H-bond acceptor parameters for the ester and amide groups (*β* = 5.3 and 7.8) are much lower than the corresponding parameter for phosphine oxide (*β* = 10.7), so H-bonding interactions between the phenol groups and the backbone carbonyl groups do not compete with the base-pairing interactions in the duplex.^32^ These observations suggest that although the primed template is in equilibrium between a folded and extended conformation, when the phosphine oxide 3-mer is added, the pre-ZIP complex is fully assembled with three H-bonds between the phosphine oxide and phenol groups.

**Fig. 7:**
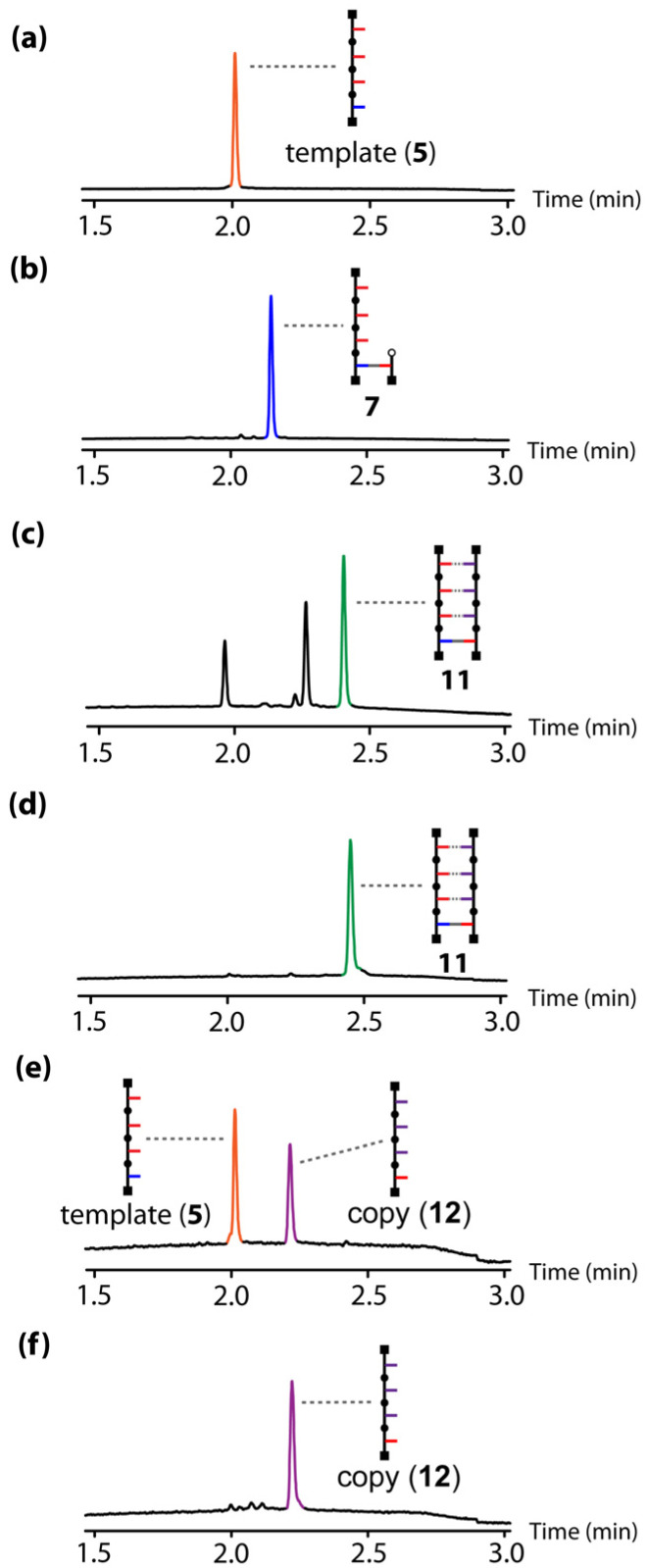
UPLC traces for H-bond template-directed oligomer synthesis using a covalent primer: (a) starting template 5; (b) primed template 7; (c) crude reaction mixture obtained after the CuAAC reaction of 7 in the presence of equimolar amounts of 8 and 9 (the additional peaks correspond to unreacted 8 and 9); (d) isolated duplex 11 (e) crude reaction mixture obtained after hydrolysis of the ester base-pair; (f) isolated copy 12. UPLC conditions: C18 column at 40 °C (254 nm) using water + 0.1% formic acid (A) and CH_3_CN + 0.1% formic acid (B); gradient of 0–2 min 5%–100% B + 1 min 100% B.

**Scheme 3:**
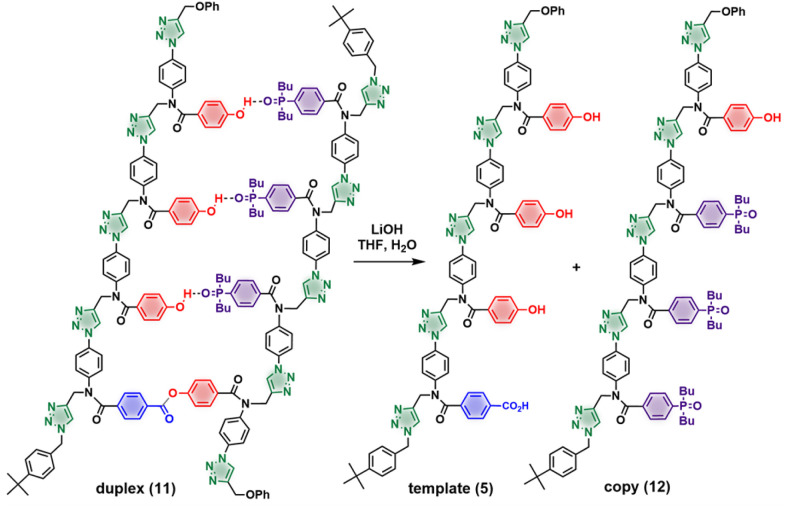
Hydrolysis of duplex 11 to yield the starting template 5 and complementary copy strand 12.

These results show that it is possible to tune the efficiency of a templating process using the strength of the interaction between the monomer and the template. The 3-mer phosphine oxide that makes three H-bonds with the primed template is therefore suitable to carry out the full replication cycle illustrated in Fig. 1. Fig. 7 shows the UPLC results for each step of the pathway starting from template 5. The first step involves covalent attachment of the primer 6 by ester coupling to give the primed template 7 (Fig. 7b). The template-directed CuAAC reaction was then carried out using a mixture of the complementary phosphine oxide 3-mer 8 and competing phenol 9 (Fig. 7c shows the UPLC trace of the crude reaction mixture). Column chromatography was used to separate duplex 11 from the excess azides (Fig. 7d). The final step is cleavage of the covalent ester base-pair to release the template and the copy strand (Scheme 3 and Fig. 7e). The two oligomers were separated by column chromatography (Fig. 7f), and the pure copy 12 was fully characterized (see ESI†).

## Conclusions

Current directed evolution methodologies rely exclusively on nucleic acids for the transfer of sequence information, limiting the scope to systems that interface with DNA. The use of synthetic polymers in evolutionary searching would give access to a vast and unexplored region of chemical structure space, opening the way to develop synthetic systems that could rival the functional properties of biopolymers. However, methods for the template-directed replication of synthetic oligomers are required before evolutionary optimization of functional polymers becomes possible. Here we describe an efficient process for template-directed replication of triazole oligomers using a combination of covalent and non-covalent base-pairing interactions to transfer information from the template to the daughter strand. A key feature is the use of a covalent primer, which is attached to the template *via* ester base-pairing, and functionalized with an alkyne group. The template has phenol recognition units able to form non-covalent base-pairs with phosphine oxide monomers *via* H-bonding. These phosphine oxide monomers are functionalized with an azide group, so the templated elongation of the primer is viable through CuAAC. Competition reactions between azides equipped with phosphine oxide recognition units and azides equipped with phenol recognition units were used to assess the template effect.

The efficiency of the templated reaction can be optimized by tuning a single parameter: the binding strength of the azide-functionalized phosphine oxide monomer. When the number of H-bonds between the template phenol units and the phosphine oxide monomer is increased, there is a significant acceleration of the templated reaction. Thus, a phosphine oxide 2-mer leads to a higher rate acceleration than a phosphine oxide 1-mer, because it binds the template with an order of magnitude higher affinity. When a 3-mer phosphine oxide strand was used, the higher affinity for the template leads to quantitative templated primer elongation.

Evidence for the phenol-phosphine oxide H-bonding involved in the templated pathway was obtained from NMR spectroscopy and is supported by molecular models. These data also suggest that the primed template is in equilibrium between a folded and extended conformation. When the phosphine oxide 3-mer is added, the template-substrate complex is fully assembled with three H-bonds between phosphine oxide and phenol groups, enabling the alignment of the azide and alkyne groups in a favourable geometry for the CuAAC reaction. The results show that the covalent and non-covalent base-pairing motifs used here are geometrically compatible and can be successfully deployed in combination.

This system was used to carry out a full cycle of templated-directed primer elongation. Starting from the mixed sequence template, the primer was covalently loaded, then templated oligomer synthesis provided the hybrid covalent/non-covalent duplex. Cleavage of the ester base-pair in the duplex regenerated the template and released the sequence-complementary copy oligomer. The behaviour of this synthetic system is in line with observations on non-enzymatic nucleic acid replication, where the rate and fidelity of primer extension can be improved by using activated oligomers as the building blocks instead of monomers.^29^

We have previously demonstrated sequence-selective oligomer replication using covalent base-pairing.^21^ Covalent base-pairs are formed in a quantitative manner, guaranteeing full loading of monomers onto a template, and they also provide a mechanism for irreversible cleavage of the product duplex. However, replication cycles using this approach require six chemical steps. The dynamic nature of non-covalent base-pairs means that base-pair formation and cleavage can be carried out avoiding many of these chemical steps, but incomplete binding to the template lead to low yields of templated product.^28^ The 3-mer building blocks introduced here form three cooperative non-covalent base-pairs, which leads to full loading of monomers onto the template and high yields of templated product. When used in conjunction with a covalent primer, the product duplex can then be cleaved to irreversibly release the daughter strand from the template. These components therefore set the scene for sequence-selective information transfer between synthetic polymers using non-covalent base-pairing.

## Author contributions

The manuscript was written through contributions of all authors.

## Conflicts of interest

There are no conflicts to declare.

## Supplementary Material

QO-010-D3QO01717F-s001
