# Supplementary material for: Replication of synthetic recognition-encoded oligomers by ligation of trimer building blocks
Source: Org Chem Front. 2023 Oct 25;10(23):5950–7. doi: 10.1039/d3qo01717f (PMC10661083; doi:10.1039/d3qo01717f)
Supplement: QO-010-D3QO01717F-s001 [file QO-010-D3QO01717F-s001.pdf]

Electronic Supplementary Material (ESI) for Organic Chemistry Frontiers.
This journal is © the Partner Organisations 2023

Supporting information

Replication of synthetic recognition-encoded oligomers by ligation of trimer building blocks

Diego Núñez-Villanueva,[a,b] Christopher A. Hunter[a]*

[a]*Yusuf Hamied Department of Chemistry, University of Cambridge, Lensfield Road, Cambridge CB2 1EW, UK.*

[b]*Present address: ComFuturo Fellow, Instituto de Química Médica (IQM-CSIC), Juan de la Cierva 3, 28006 Madrid, Spain.*

E-mail: herchelsmith.orgchem@ch.cam.ac.uk

TABLE OF CONTENTS **Page**

1. General experimental details. S2

2. Synthesis and characterization of template and primed template S3

3. Template-directed synthesis S11

4. Hydrolysis of duplex 11 providing copy 12 S18

5. Full replication cycle from template 5 S23

6. Molecular modelling S25

7. References S26

1. General experimental details.

All the reagents and materials used in the synthesis of the compounds described below were bought from commercial sources, without prior purification. Dry THF and CH_2Cl_2 were obtained from a solvent purification system (Pure Solv™, Innovative Technology, Inc.). Anhydrous DMF was purchased from Sigma-Aldrich. Thin layer chromatography was carried out using with silica gel 60F (Merck) on glass plates. Flash chromatography was carried out on an automated system (Combiflash Rf+ or Combiflash Rf Lumen) using prepacked cartridges of silica (25µ PuriFlash® columns). All NMR spectroscopy was carried out on a Bruker 400 MHz DPX400, 400 MHz AVIII400, 500 MHz DCH cryoprobe or 500 MHz TCI Cryoprobe spectrometer using the residual solvent as the internal standard. All chemical shifts (δ) are quoted in ppm and coupling constants given in Hz. Splitting patterns are given as follows: s (singlet), bs (broad singlet), d (doublet), t (triplet), q (quadruplet), m (multiplet). FT-IR spectra were measured on a Bruker Alpha spectrometer equipped with an ATR cell. UPLC analysis of samples was performed using Waters Acquity H-class UPLC coupled with a single quadrupole Waters SQD2. Acquity UPLC CSH C18 column, 130 Å, 1.7 µm, 2.1 mm x 50 mm or Acquity UPLC BEH C8 column, 130 Å, 1.7 µm, 2.1 mm x 50 mm were used as UPLC columns. The conditions of the UPLC method are as follows: gradients of water + 0.1% formic acid (solvent A) and acetonitrile + 0.1% formic acid (solvent B) as specified in each case. Flow rate: 0.6 ml/min; Column temperature of 40°C; Injection volume of 2 µL. The signal was monitored at 254 nm. HRMS analysis was performed in an Agilent walk up 6230 LC/TOF using a gradient from 5 to 100% of acetonitrile (0.25% formic acid) in water (0.25% formic acid) over 6 minutes.

Compounds **1**[S1], **2**[S1], **4**[S1], **6**[S1], **8**[S2] and **9**[S2] have been previously described.

2. Synthesis and characterization of template and primed template

Synthesis of 4-mer template and loading of primer.

The synthesis of the 4-mer template and the corresponding primer-loaded derivative is highlighted in Scheme S1. From 2-mer **1**[S1], the CuAAC coupling with protected monomer **2**[S1] and subsequent TBAF-mediated deprotection yielded 3-mer **3**. CuAAC coupling of **3** with monomer **4**[S1] gave rise to 4-mer template **5**. Primer **6**[S1] was directly loaded onto the template by ester coupling and the primer-loaded derivative **7** was obtained in low yield due to the preferential formation of the corresponding lactone.

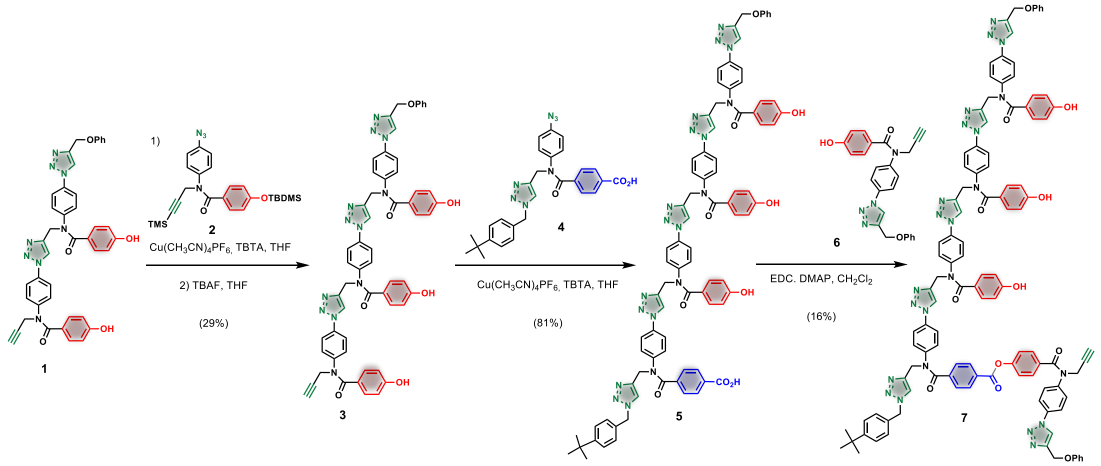

Scheme S1. Synthesis of 4-mer template **5** and primer-loaded template **7**.

Compound 3

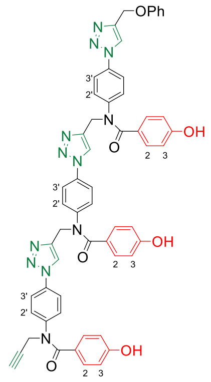

Compound **1**[S1] (0.052 g, 0.07 mmol), compound **2**[S1] (0.035 g, 0.07 mmol), $Cu(CH_3CN)_4PF_6$ (0.003 g, 0.007 mmol) and TBTA (0.004 g, 0.007 mmol) were mixed in a round-bottom flask and, under N_2, THF (10 mL) was added. The reaction was stirred overnight at room temperature. Once the reaction was completed, TBAF (1M in THF, 0.140 mL, 0.14 mmol) was added dropwise. The solution was stirred for 10 min at room temperature, quenched with 5% soln. HCl and extracted with EtOAc (3x) followed by washing with H_2O and brine. The organic layer was dried over anhydrous $MgSO_4$ and concentrate under vacuum. The crude was purified by flash column chromatography on a C18 reverse phase column (gradient from 5% to 100% of CH_3CN in H_2O) to afford **3** (0.021 g, 29%) as a amorphous white solid.

^{1}H NMR (500 MHz, DMSO-*d*$_6$): δ_H 8.87 (s, 1H, CH_{triaz}, PhO cap), 8.71 and 8.68 (s, 1H, CH_{triaz}, internal), 7.85 (d, 2H, *J* = 9.0 Hz, 3'-H), 7.78 (d, 4H, *J* = 9.0 Hz, 3'-H), 7.40 and 7.39 (d, 2H, *J* = 9.0 Hz, 2'-H), 7.33 (d, 2H, *J* = 9.0 Hz, 2'-H), 7.29 (m, 2H, 3''-H), 7.21 (d, 2H, *J* = 9.0 Hz, 2-H), 7.18 (d, 2H, *J* = 9.0 Hz, 2-H), 7.15 (d, 2H, *J* = 9.0 Hz, 2-H), 7.05 (d, 2H, *J* = 8.0 Hz, 2''-H), 6.95 (t, 2H, *J* = 7.5 Hz, 4''-H), 6.58 (d, 2H, *J* = 8.5 Hz, 3-H), 6.55 (d, 4H, *J* = 8.5 Hz, 3-H), 5.19 (s, 2H, O-CH_2), 5.18 (s, 2H, N-CH_2, phenol), 5.16 (s, 2H, N-CH_2, phenol), 4.65 (d, 2H, *J* = 2.5 Hz, N-CH_2, terminal), 3.15 (t, 1H, *J* = 2.5 Hz, alkyne).

^{13}C NMR: Not recorded due to limit in available material.

HRMS (ES+): calcd for $C_{57}H_{44}N_{12}O_7$ 1031.3354 $[M+Na]^+$, found 1031.3284 $[M+Na]^+$.

FT-IR (ATR): ν_{max} 3241, 1962, 2953, 2925, 1633, 1607, 1517, 1279, 1237, 1170 and 845 cm^{-1}.

^{1}H-NMR (500 MHz, DMSO-d_6) compound 3

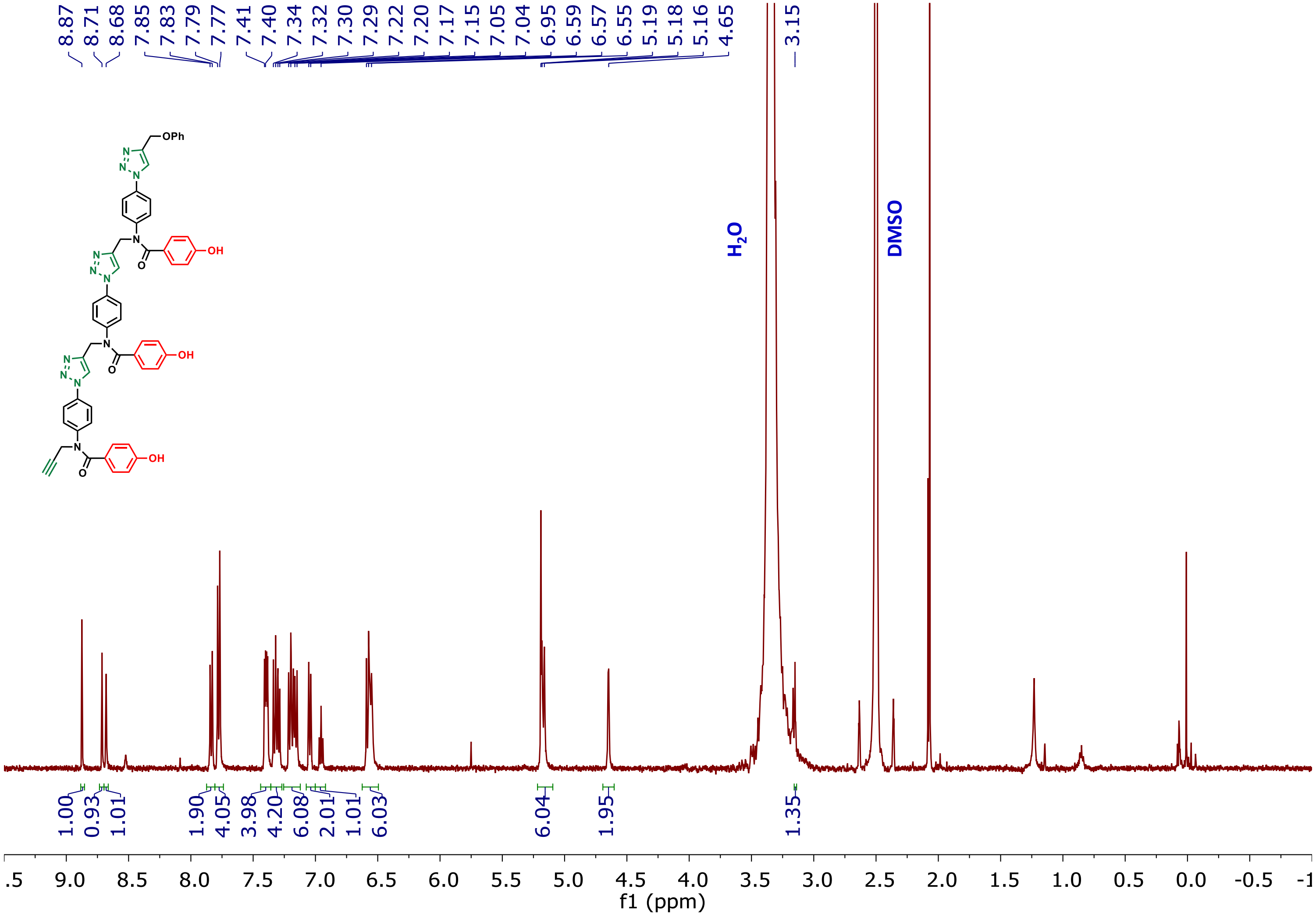

Template 5

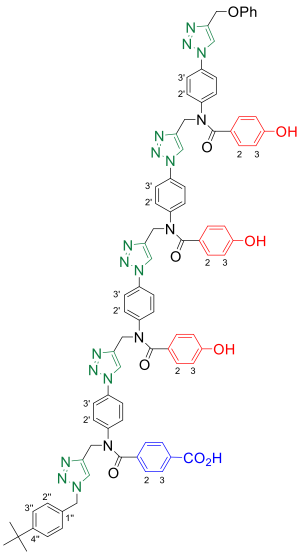

Compound **3** (0.020 g, 0.02 mmol), compound **4** (0.011 g, 0.02 mmol), $Cu(CH_3CN)_4PF_6$ (0.004 g, 0.01 mmol) and TBTA (0.005 g, 0.01 mmol) were mixed in a round-bottom flask and, under N_2, THF (5 mL) was added. The reaction was stirred overnight at room temperature. The reaction was then diluted with EtOAc and washed with EDTA soln. (2x), H_2O (1x) and brine. The organic layer was dried over $MgSO_4$ and concentrate under vacuum. The residue was purified by flash column chromatography on silica gel (gradient from 0% to 18% of MeOH in CH_2Cl_2) to afford **5** (0.023 g, 81%) as a amorphous white solid.

^{1}H NMR (500 MHz, DMSO-*d*$_6$): δ_H = 9.86 (s, 3H, OH), 8.87 (s, 1H, CH_{triaz}, PhO cap), 8.69, 8.69 and 8.68 (s, 2H, CH_{triaz}, internal), 8.09 (s, 1H, CH_{triaz}, ^{t}Bu cap), 7.76 (m, H, 3-H, acid; 3'-H), 7.41 (d, 2H, *J* = 9.0 Hz, 2'-H), 7.37 (d, 4H, *J* = 9.0 Hz, 2'-H), 7.34 (d, 4H, *J* = 9.0 Hz, 2'-H), 7.33 (d, 2H, *J* = 9.0 Hz, 2-H, acid), 7.29 (d, 2H, *J* = 9.0 Hz, 3''-H, PhO cap), 7.27 (d, 2H, *J* = 9.0 Hz, 3''-H, ^{t}Bu cap), 7.20 (m, 6-H, phenol), 7.08 (d, 2H, *J* = 8.5 Hz, 2''-H, ^{t}Bu cap), 7.05 (d, 2H, *J* = 8.0 Hz, 2''-H, PhO cap), 6.95 (t, 1H, *J* = 7.5 Hz, 4''-H, PhO cap), 6.58 (m, 6H, 3-H, phenol), 5.49 (s, 2H, N-CH_2, ^{t}Bu cap), 5.19 (s, 2H, PhO cap), 5.17 (s, 2H, N-CH_2), 5.15 (s, 6H, N-CH_2), 1.16 (s, 9H, ^{t}Bu).

^{13}C NMR (125 MHz, DMSO-*d*$_6$): δ_C = 169.3, 169.2 and 169.2 (CO, amide phenol), 168.8 (CO, amide acid), 167.0 (CO, acid), 159.1 and 159.0 (4-C, phenol), 157.9 (1''-C, PhO cap), 150.5 (4''-C, ^{t}Bu cap), 144.7, 144.6, and 143.9 (C_{triaz}), 143.9, 147.8 and 143.7 (1'-C), 142.9 (C_{triaz}), 142.9 (1'-C), 142.3 (1-C, acid), 134.6, 134.2, 134.2 and 134.2 (4'-C), 133.2 (4-C, acid; 1''-C, ^{t}Bu cap), 130.9 and 130.9 (2-C, phenol), 129.6, 129.2, 128.8, 128.8, 128.8, 128.7, 128.3, 128.2 and 127.3 (C_{arom}), 125.7, 125.6 and 125.5 (1-C, phenol), 125.4 (3''-C, ^{t}Bu cap), 124.1 (CH_{triaz}, ^{t}Bu cap) 122.8 (CH_{triaz}, PhO cap), 121.6 and 121.6 (CH_{triaz}, phenol), 121.5 (CH_{triaz}, acid), 121.0 (4''-C, PhO cap), 120.6, 120.5, 120.4 and 120.3 (3'-C), 114.7 (2''-C, PhO cap), 114.6, 114.5 and 114.5 (3-C, phenol), 60.9 (O-CH_2), 52.4 (N-CH_2, ^{t}Bu cap), 45.5, 45.4 and 45.4 (N-CH_2), 34.2 (C, ^{t}Bu), 31.0 (CH_3, ^{t}Bu).

HRMS (ES+): calcd for $C_{85}H_{71}N_{19}O_{10}$ 1518.5710 $[M+H]^+$, found 1518.5716 $[M+H]^+$.

FT-IR (ATR): ν_{max} 3446, 2957, 2923, 1638, 1606, 1518, 1432, 1281, 1239 and 846 cm^{-1}.

^{1}H-NMR (500 MHz, DMSO-d_6) compound 5

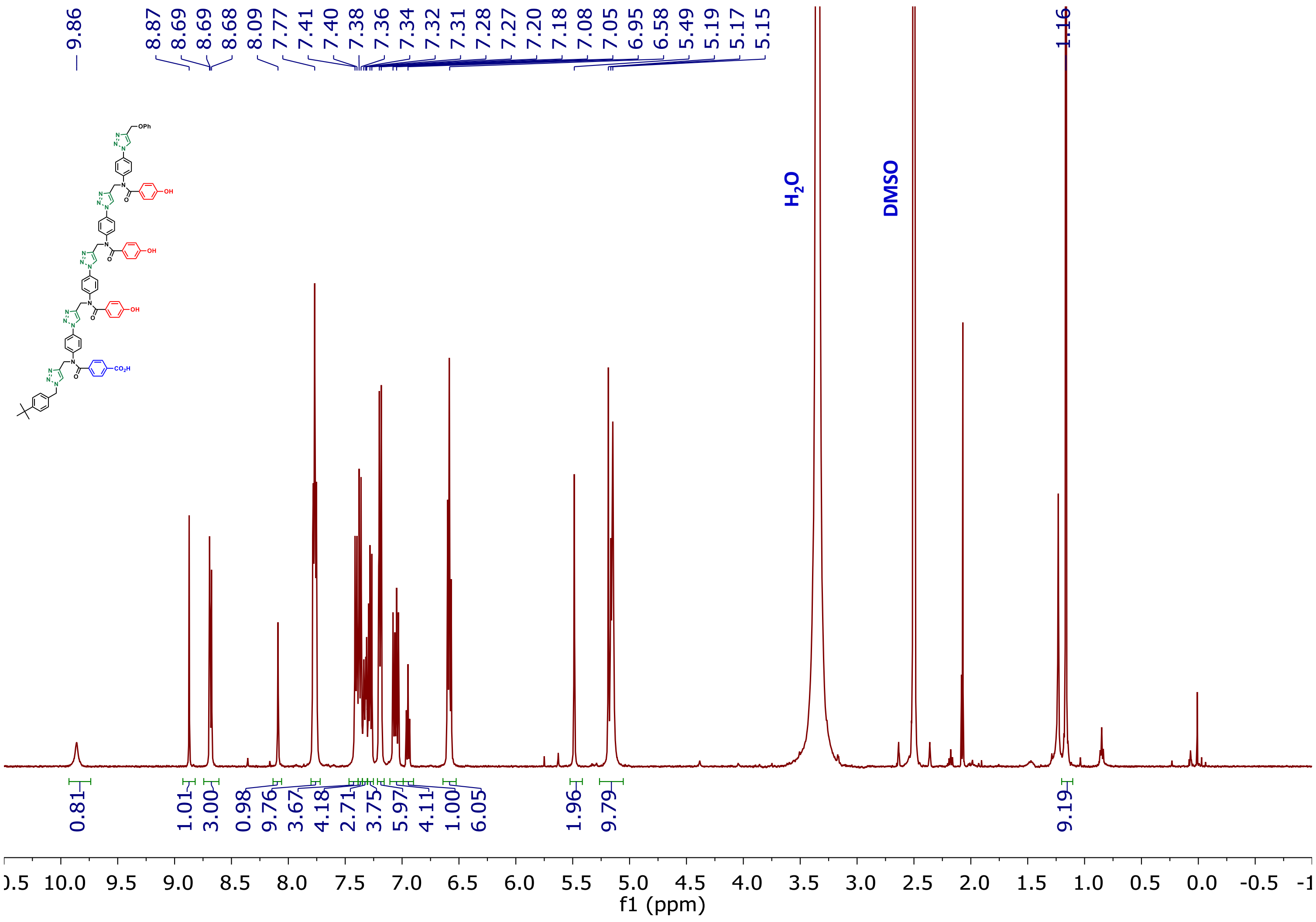

^{13}C-NMR (125MHz, DMSO-d_6) compound 5

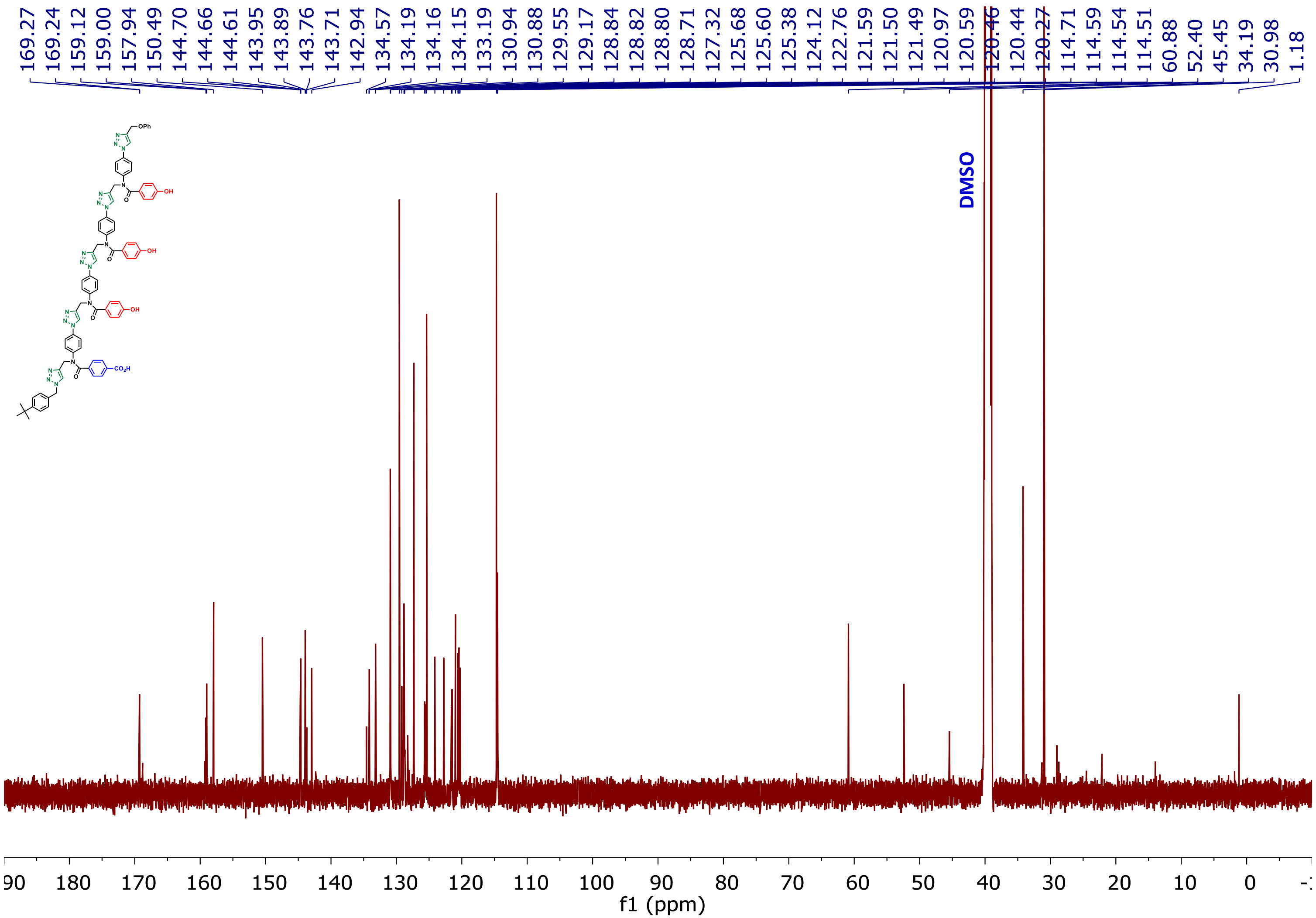

Primed template 7

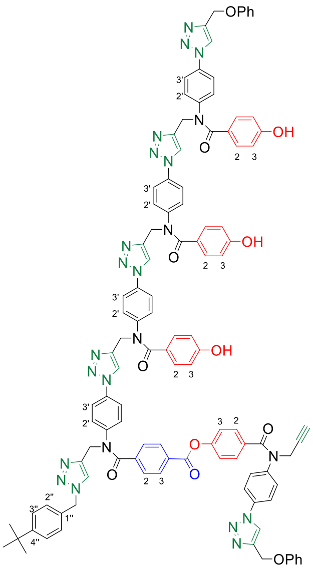

Compound **5** (0.015 g, 0.01 mmol), primer **6** (0.032 g, 0.008 mmol), EDC (0.004 g, 0.02 mmol) and DMAP (0.002 g, 0.02 mmol) were dissolved in dry CH_2Cl_2 (1 mL) under N_2 atmosphere. The reaction was stirred overnight at room temperature. Once finished, the reaction was diluted with EtOAc and washed with 0.1N HCl soln. (2x), H_2O (2x) and brine (1x). The solution was dried with anhydrous $MgSO_4$, filtered and the solvent evaporated. The obtained residue was purified by flash chromatography (gradient from 0% to 65% of EtOAc in pet. ether followed by a gradient from 0% to 18% of MeOH in CH_2Cl_2) to afford **7** (0.003 g, 16%) as a white amorphous solid.

^{1}H NMR (500 MHz, $CDCl_3$): δ_H = 8.14 (s, 1H, CH_{triaz}, internal), 8.11 (s, 1H, CH_{triaz}, internal), 8.01 (s, 1H, CH_{triaz}, PhO cap), 7.99 (s, 1H, CH_{triaz}, PhO cap), 7.90 (d, 2H, J = 8.5 Hz, 3-H, ester), 7.68 (s, 1H, CH_{triaz}, ^{t}Bu cap), 7.63 (d, 2H, J = 9.0 Hz, 3'-H), 7.55 (s, 1H, CH_{triaz}, internal), 7.52 (m, 8H, 3'-H), 7.37 (m, 6H, 2-H, primer; 2-H, ester; 3''-H, ^{t}Bu cap), 7.26 (m partially overlapped, 14H, 2'-H; 3''-H, PhO caps), 7.19 (d, 2H, J = 8.5 Hz, 2''-H, ^{t}Bu cap), 7.14 (d, 2H, J = 8.5 Hz, 2-H, phenol), 7.12 (d, 2H, J = 8.5 Hz, 2-H, phenol), 7.09 (d, 2H, J = 8.5 Hz, 2-H, phenol), 7.01 (d, 2H, J = 6.5 Hz, 3-H, primer), 6.95 (m, 6H, 2''-H and 4''-H, PhO cap), 6.55 (m, 6H, 3-H, phenol), 5.47 (s, 2H, N-CH_2, ^{t}Bu cap), 5.23 (s, 4H, O-CH_2, PhO cap), 5.19 (s, 2H, N-CH_2, internal), 5.15 (s, 2H, N-CH_2, internal), 5.13 (s, 2H, N-CH_2, internal), 5.11 (s, 2H, N-CH_2, terminal), 4.67 (d, 2H, J = 2.5 Hz, N-CH_2, alkyne), 2.27 (t, 1H, J = 2.5 Hz, CH, alkyne), 1.29 (s, 9H, ^{t}Bu).

^{13}C NMR (125.7 MHz, $CDCl_3$): Not recorded due to limit in available material.

HRMS (ES+): calcd $C_{110}H_{89}N_{23}O_{12}$ 1924.7134 $[M+H]^+$, found 1924.7100 $[M+H]^+$.

FT-IR (ATR): ν_{max} 2957, 2920, 2870, 1739, 1636, 1605, 1517, 1261, 1237 and 1043 cm^{-1}.

^{1}H-NMR (500 MHz, $CDCl_3$) compound 7

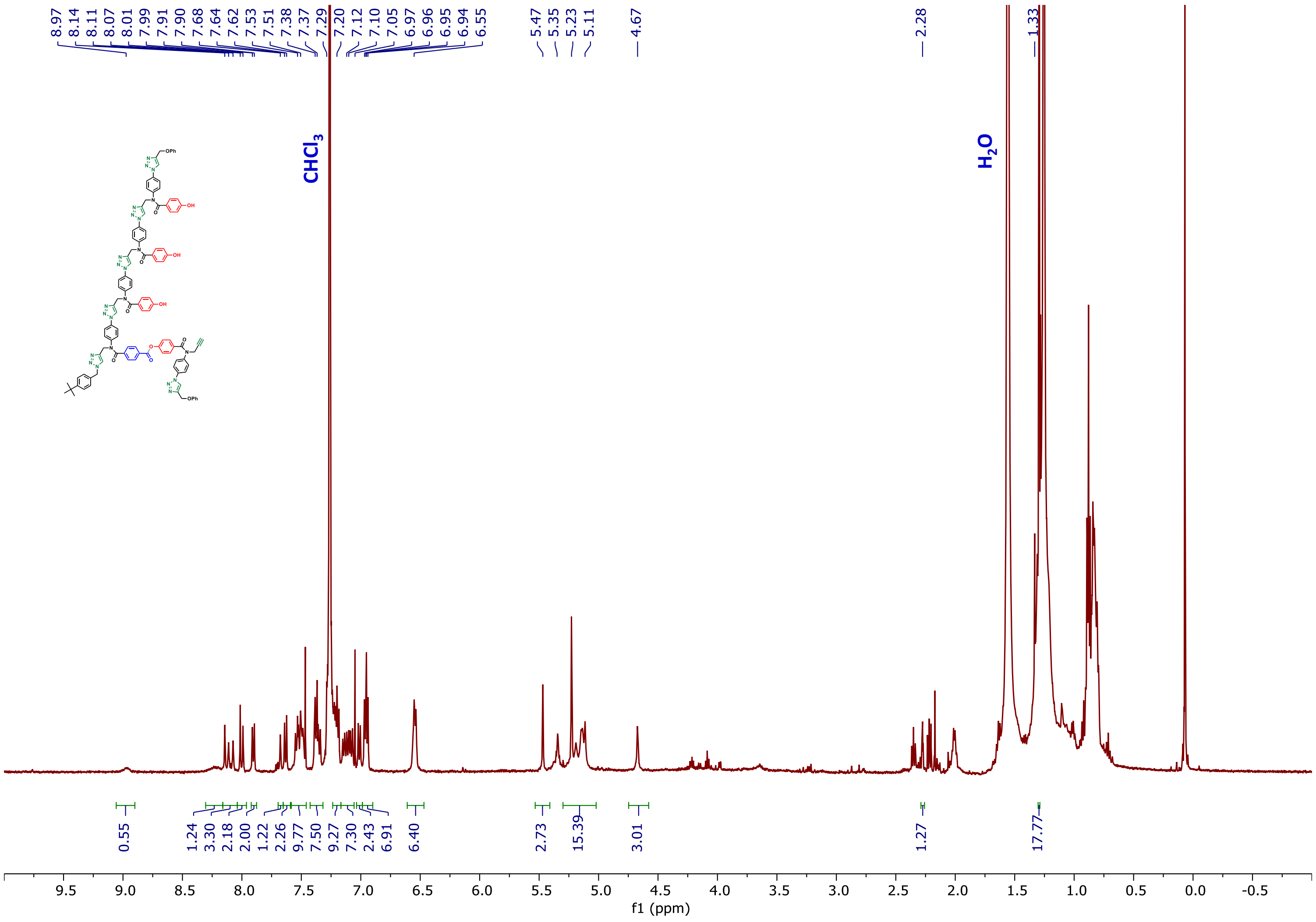

3. Templating reaction using primed template 7, 3-mer 8 and 1-mer 9

Compound **7** is used as template for the template-directed synthesis of its complementary duplex from the alkyne functionality provided by the primer. Two different azides are used: **8** is the complementary phosphine oxide 3-mer while **9** is the non-complementary one. CuAAC reaction with **7** leads to the formation of two products: **10** is the non-templated product from the reaction of **7** and **9** while **11** is the templated product from the reaction of **7** and **8** (Scheme S2. The template effect provided by H-bonding between the phenol groups in the template and the phosphine oxides of the 3-mer is studied using the simple alkyne **S1** as control (Scheme S3).

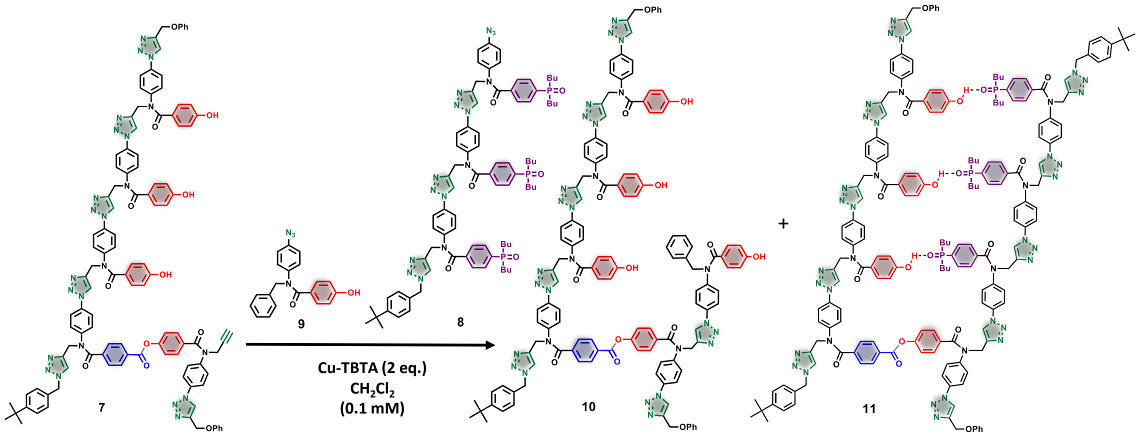

Scheme S2. Reaction schemes for the template-directed synthesis of the sequence complementary duplex from **7** using 1-mer **9** and 3-mer **8**.

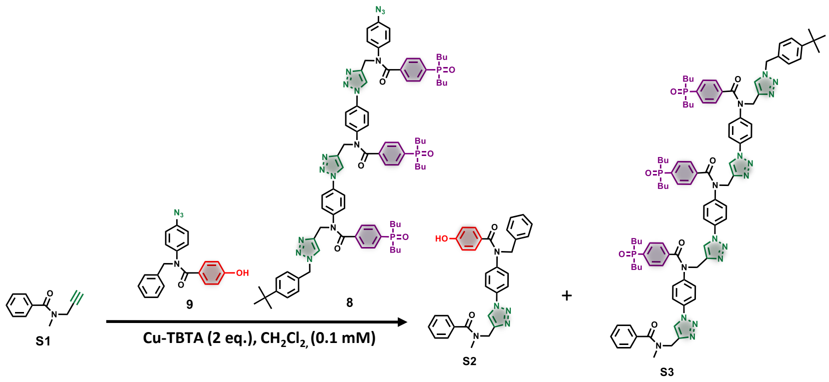

Scheme S3. Control reaction where no template effect is possible.

General procedure for the templating and control reactions shown in schemes S2 and S3.

From freshly prepared stock solutions in dry CH_2Cl_2, the starting alkyne (**7** or **S1**, $3.4{\cdot}10^{-5}$ mmol), **9** (0.017 mg, $5.1{\cdot}10^{-5}$ mmol) and **8** (0.085 mg, $5.1{\cdot}10^{-5}$ mmol) were mixed in a 1.75 mL vial containing a magnetic stirrer. The solvent was evaporated under N_2 stream and dry CH_2Cl_2 (0.34 mL) was added. A 30 µL aliquot was taken for UPLC analysis (t_0). To this solution, 10 µL of a stock solution of premixed $Cu(CH_3CN)_4PF_6$ (2.2 mg, $6.8{\cdot}10^{-3}$ mmol) and TBTA (3.2 mg, $6.8{\cdot}10^{-3}$ mmol) in dry CH_2Cl_2 (1 mL) was added. The vial was flushed briefly with N_2, sealed and left stirring at room temperature for 2 days. Another 30 µL aliquot was taken after this time for

UPLC analysis (t_f). Figure S1 shows the UPLC traces for the templating and control experiments. Table S1 includes the peak areas from these chromatograms for phenol **9** and phosphine oxide **8** before (t_0) and after (t_f) Cu-TBTA was added along with the calculated *k'*.[S2]

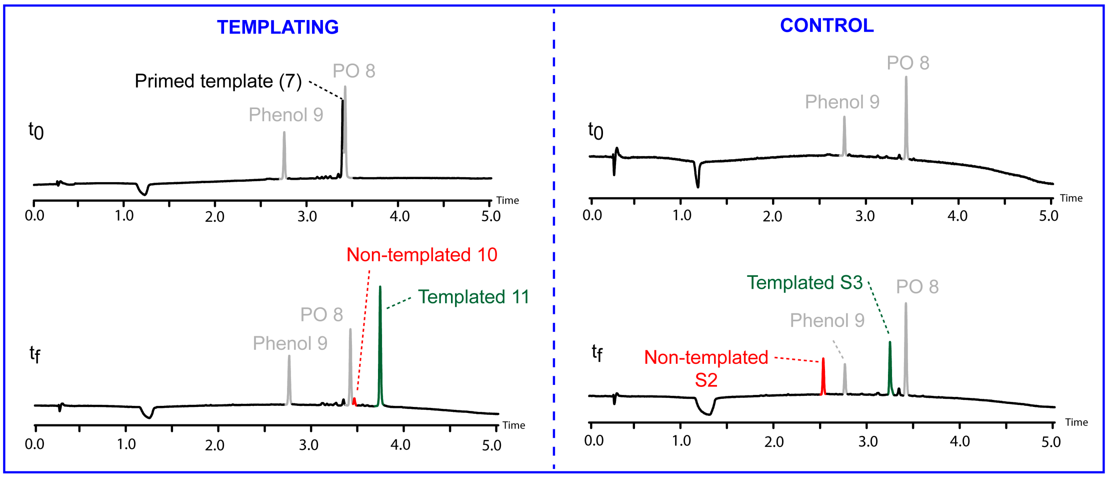

Figure S1. UPLC traces of the templating (left, scheme S2) and control (right, scheme S3) reactions before (t_0) and after (t_f) Cu-TBTA was added. All the peaks are labelled: Phenol (compound **9**), PO (compound **8**), primed template (compound **7**), non-templated (compound **10** for the templating reactions on the left; compound **S2** for the control reactions on the right) and templated (compound **11** for the templating reactions on the left; compound **S3** for the control reactions on the right). *Conditions:* C18 column at 40 °C (270 nm) using water + 0.1% formic acid (A) and CH_3CN + 0.1% formic acid (B) with flow rate of 0.6 ml/min; Gradient of 0-4 min 5% -100% B + 1 min 100% B.

Table S1. Peak areas from the UPLC chromatograms shown in Figure S1 for phenol **9** and phosphine oxide **8** before (t_0) and after (t_f) Cu-TBTA was added along with calculated *k'*.

		Phenol 9	Phosph. ox. 8	χ	*k*	*k'*
**Templating**	t_0	1415	2877	1.62	2.79	5.44
	t_f	1190	1495			
**Control**	t_0	462	818	0.67	0.43	
	t_f	929	2454			

Synthesis of templated product 11 (for characterization purposes)

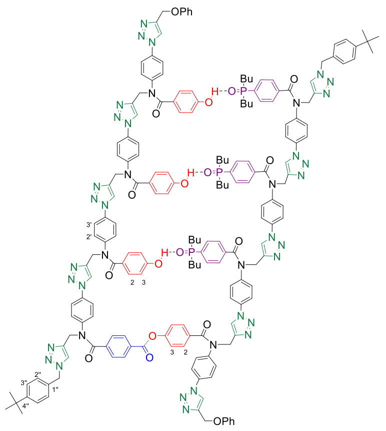

A solution of **7** (2.5 mg, 1.3·10^{-3} mmol) and **8** (2 mg, 1.3·10^{-3} mmol) in CH_2Cl_2 (2.5 mL) under N_2 atmosphere was treated with $Cu(CH_3CN)_4PF_6$ (2 mg, 5.2·10^{-3} mmol) and TBTA (3 mg, 5.2·10^{-3} mmol). The solution was stirred overnight at room temperature. The reaction was then diluted with EtOAc and washed with EDTA soln. (2x), H_2O (1x) and brine. The organic layer was dried over $MgSO_4$ and concentrate under vacuum. The residue was purified by flash column chromatography on C18 reverse phase (gradient from 10% to 85% of CH_3CN in H_2O) to afford **11** (1 mg, 30%) as a white amorphous solid. UPLC traces shown below correspond to: (a) the starting materials; (b) the obtained reaction crude and (c) the isolated duplex **11**. *UPLC Conditions*: C18 column at 40 °C (254 nm) using water + 0.1% formic acid (A) and CH_3CN + 0.1% formic acid (B) with flow rate of 0.6 ml/min; Gradient of 0-2 min 5% -100% B + 1 min 100% B.

(a) Starting materials

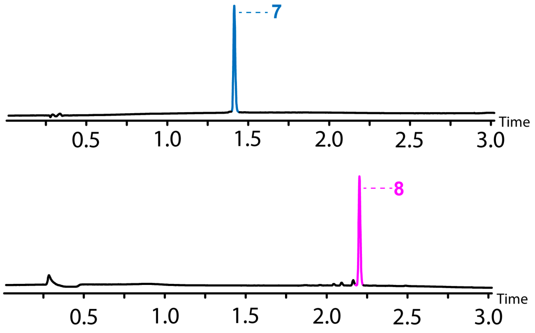

(b) Reaction crude

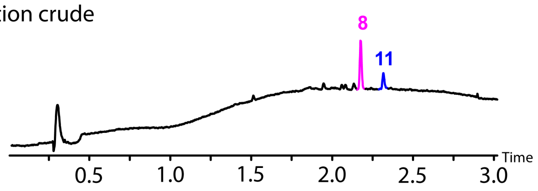

(c) Compound **11** (isolated)

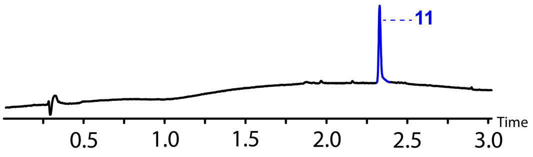

Characterization of compound 11.

^{1}H NMR (500 MHz, $CDCl_3$): δ_H = 10.2 (s, 1H, OH), 9.92 (s, 1H, OH),), 9.76 (s, 1H, OH), 8.27 (s, 1H, CH_{triaz}), 8.23 (s, 1H, CH_{triaz}), 8.23 (s, 1H, CH_{triaz}), 8.21 (s, 1H, CH_{triaz}), 8.20 (s, 1H, CH_{triaz}), 8.06 (s, 1H, CH_{triaz}), 8.03 (s, 1H, CH_{triaz}), 7.89 (d, 2H, *J* = 8.5 Hz, 3-H, ester), 7.67 (s, 1H, CH_{triaz}, ^{t}Bu cap), 7.66 (s, 1H, CH_{triaz}, ^{t}Bu cap), 7.60 (m, 16H, 3'-H), 7.47 (m, 6H, 3-H, PO), 7.39 (m, 10H, 2-H, PO; 3''-H, ^{t}Bu cap), 7.35-7.24 (m, 20H, 2-H, ester; 2-H, primer; 2'-H; 3''-H, PhO cap), 7.20 (d, 4H, *J* = 8.5 Hz, 2''-H, ^{t}Bu cap), 7.13 (d, 2H, *J* = 8.5 Hz, 2-H, phenol), 7.09 (m, 4H, 2-H, phenol), 7.00 (d, 4H, *J* = 8.0 Hz, 2''-H, PhO caps), 6.98 (m, 2H, 4''-H, PhO caps), 6.95 (d, 2H, *J* = 8.5 Hz, 3-H, primer), 6.58 (m, 6H, 3-H, phenol), 5.48 (s, 2H, N-CH_2, ^{t}Bu cap), 5.47 (s, 2H, N-CH_2, ^{t}Bu cap), 5.29 (s, 2H, O-CH_2, PhO cap), 5.27 (s, 2H, O-CH_2, PhO cap), 5.21 (s, 4H, N-CH_2), 5.20 (s, 4H, N-CH_2), 5.18 (s, 2H, N-CH_2), 5.17 (s, 2H, N-CH_2), 5.13 (s, 2H, N-CH_2), 5.12 (s, 2H, N-CH_2), 1.86 (m, 6H, 1''-H, Bu), 1.72 (m, 6H, 1''-H, Bu), 1.45 (m, 6H, 2''-H, Bu), 1.30 (s, 18H, ^{t}Bu), 1.23 (m, 18H, 2''-H and 3''-H, Bu;), 0.76 (t, 6H, *J* = 7.0 Hz, 4''-H, Bu), 0.72 (t, 6H, *J* = 7.0 Hz, 4''-H, Bu), 0.68 (t, 6H, *J* = 7.0 Hz, 4''-H, Bu).

^{13}C NMR (125.7 MHz, $CDCl_3$): δ_C = 170.4, 170.3, 170.2, 169.5, 169.5, 169.4, 169.3 and 169.2 (CO, amide), 163.6 (CO, ester), 160.1, 159.9 and 159.8 (4-C, phenol), 158.3 (1''-C, PhO caps), 152.2, 152.2 and 152.1 (4-C, primer; 4''-C, ^{t}Bu caps), 145.4, 145.3, 145.3, 145.2, 145.1, 145.0, 144.6, 144.3, 144.2, 144.0, 143.8, 143.8, 143.7, 143.6, 143.6, 143.5, 143.3 (1'-C; C_{triaz}), 140.4 (1-C, ester), 138.5, 138.4 and 138.3 (d, *J* = 2.5 Hz, 1-C, PO), 135.5, 135.5, 135.4, 135.0, 134.9, 134.9, 134.8, 134.7, 134.1 and 133.8 (4-C, PO; 4'-C), 132.3 (1-C, primer), 131.5 and 131.4 (1''-C, ^{t}Bu caps), 131.3, 130.5, 130.4, 130.3, 130.2, 130.2, 130.0, 129.9, 129.8, 129.2, 129.1, 129.1, 129.0, 128.9, 128.7, 128.7, 128.4, 128.3, 128.2, 128.1, 128.1 (C_{arom}), 126.3 and 126.2 (3''-C, ^{t}Bu caps), 125.5, 125.4 and 125.1 (1-C, phenol), 124.0 and 123.9 (CH_{triaz}, ^{t}Bu cap), 122.1, 122.1 and 122.1 (CH_{triaz}), 121.6, 121.4, 121.3, 121.3, 121.1, 121.1, 121.1 and 121.0 (3'-C; 4''-C, PhO caps), 121.0, 120.9, 120.9 and 120.8 (CH_{triaz}), 115.1 and 115.1 (3-C, phenol), 114.9 and 114.9 (2''-H, PhO caps), 62.0 and 62.0 (O-CH_2, PhO caps), 54.2 and 54.1 (N-CH_2, ^{t}Bu caps), 46.6, 46.5, 46.5, 46.4, 46.4, 46.3 and 46.1 (N-CH_2), 34.8 (C, ^{t}Bu), 31.4 (CH_3, ^{t}Bu), 29.3, 29.2 and 29.1 (d, *J* = 68.5 Hz, 1''-C, Bu), 24.1, 24.0 and 23.9 (d, *J* = 14.0 Hz, 2''-C, Bu), 23.5, 23.5, 23.4 and 23.4 (d, *J* = 4.0 Hz, 3''-C, Bu), 13.7, 13.6 and 13.6 (4''-C, Bu).

^{31}P NMR (161.9 MHz, $CDCl_3$): δ_P = 44.5, 44.0 and 42.9.

HRMS (ES+): calcd $C_{153}H_{151}N_{30}O_{14}P_2$ 1711.7205 $[M+2H]^{2+}$, found 1711.7222 $[M+2H]^{2+}$.

FT-IR (ATR): ν_{max} 2956, 2922, 2853, 1739, 1643, 1605, 1517, 1281, 1240, 1167 and 1043 cm^{-1}.

^{1}H-NMR (500 MHz, $CDCl_3$) compound 11

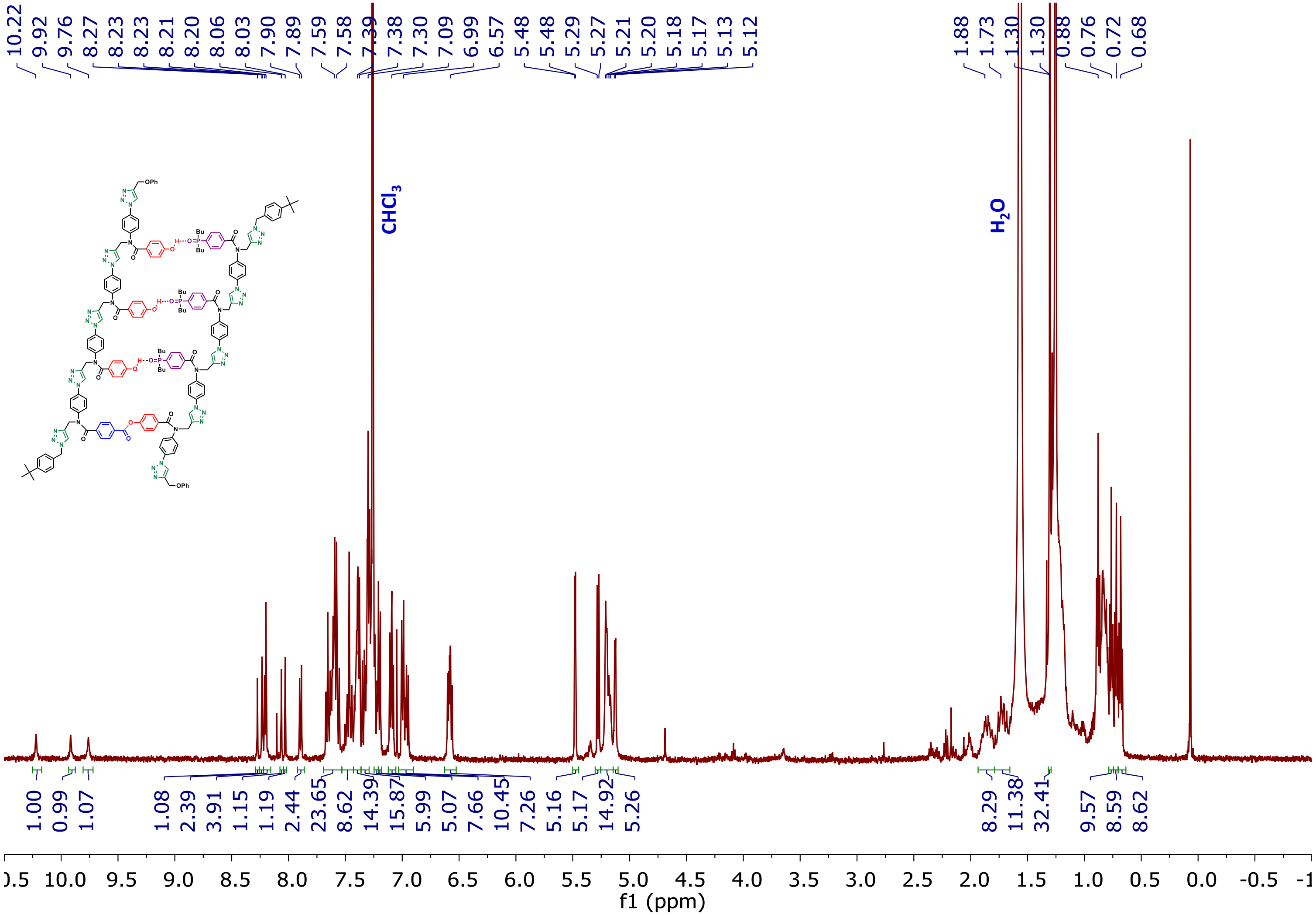

^{13}C-NMR (125.7 MHz, $CDCl_3$) compound 11

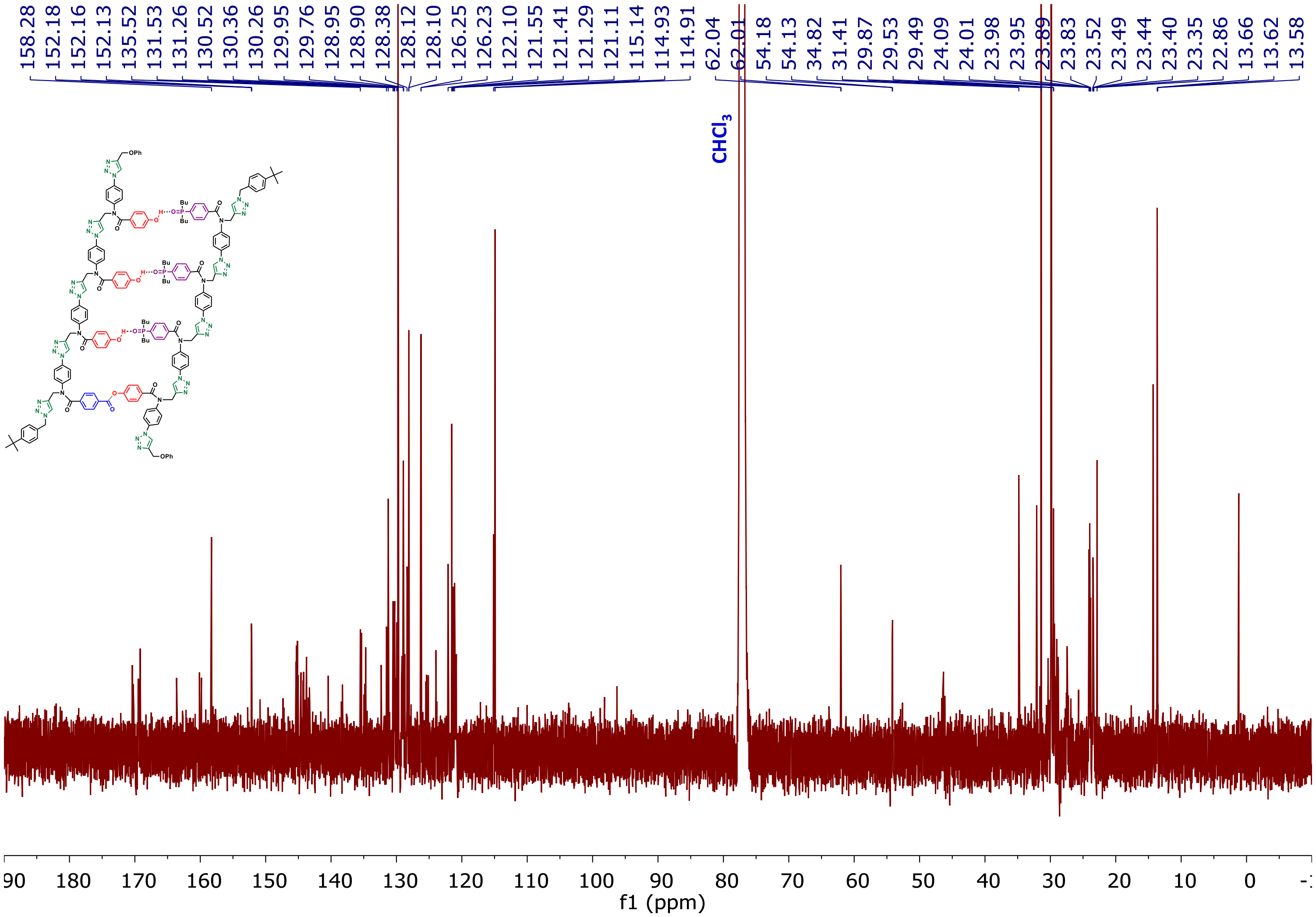

^{31}P NMR (161.9 MHz, $CDCl_3$) compound 11

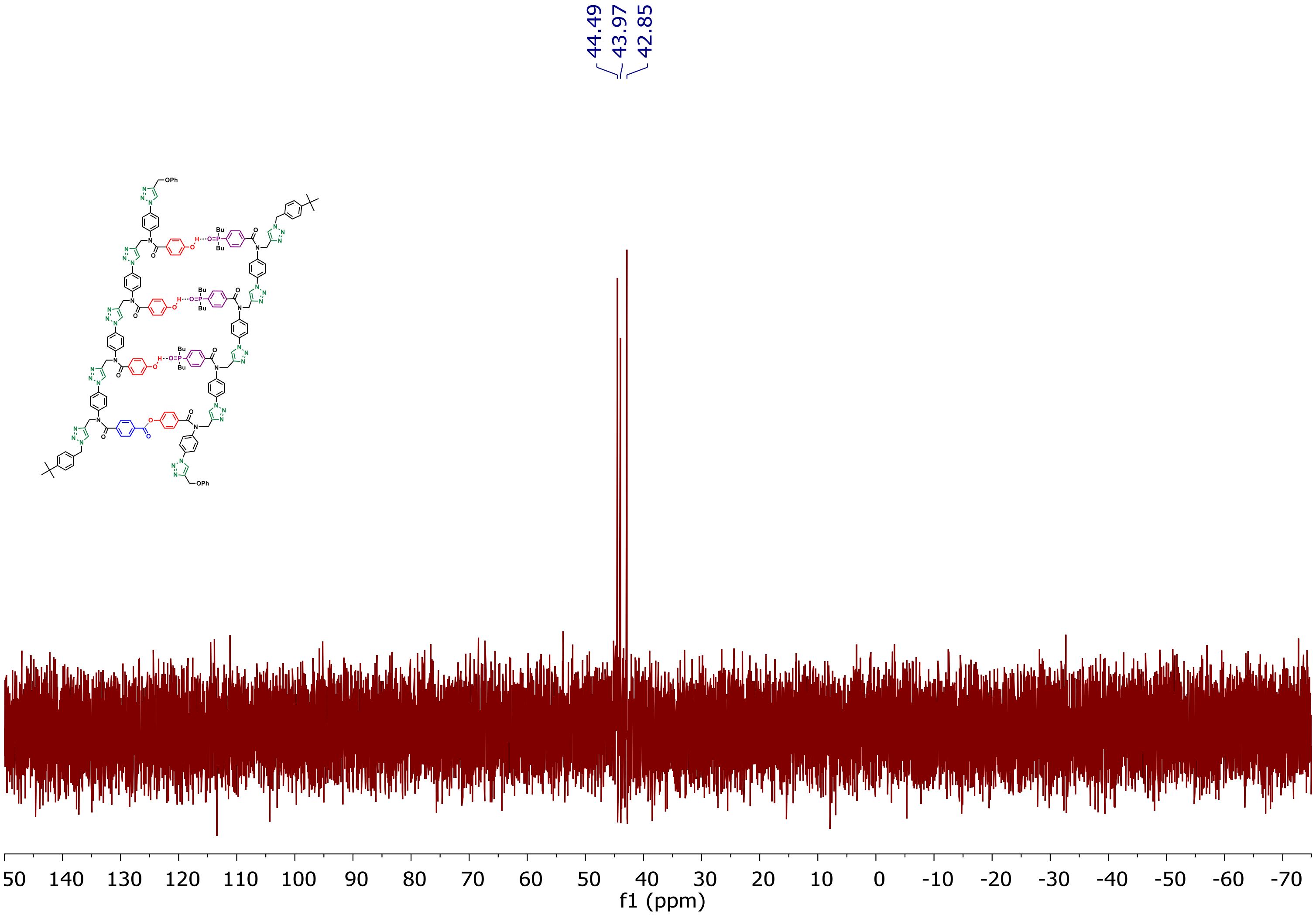

4. Hydrolysis of duplex 11 providing copy 12

Basic hydrolysis of the ester bond in duplex **11** afforded the corresponding complementary copy (**12**) along with the starting template **5**, as shown in scheme S4. Figure S2 shows the corresponding UPLC traces for the hydrolysis reaction and isolation of template and copy.

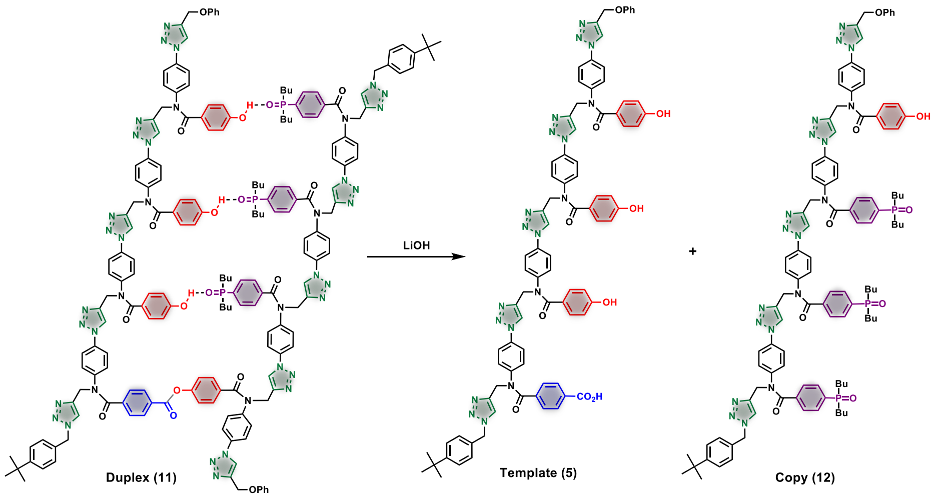

Scheme S4. Cleavage of the ester in **11** to give access to copy **12** along with template **5**.

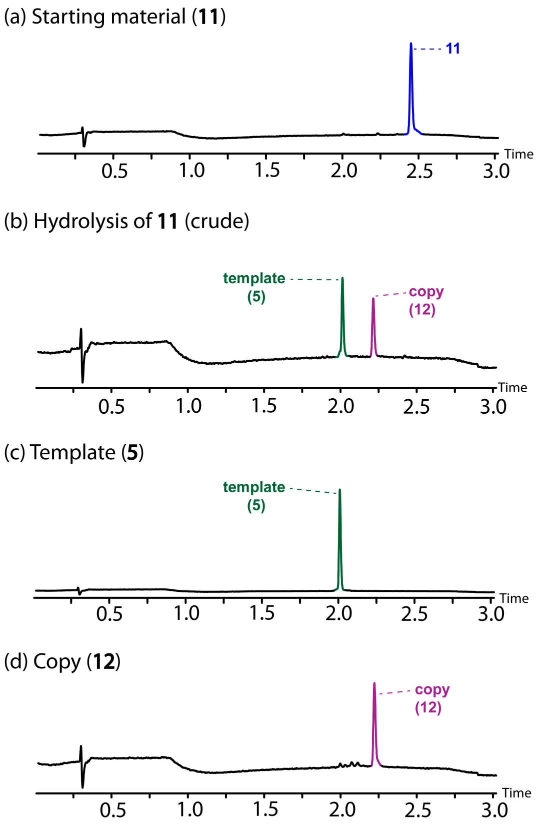

Figure S2. UPLC traces for the cleavage step: (a) the starting material; (b) the obtained reaction crude; (c) and (d) isolated template and copy strands. *UPLC Conditions*: C18 column at 40 °C (254 nm) using water + 0.1% formic acid (A) and CH_3CN + 0.1% formic acid (B) with flow rate of 0.6 ml/min; Gradient of 0-4 min 5% -100% B + 1 min 100% B.

Synthesis of copy 12.

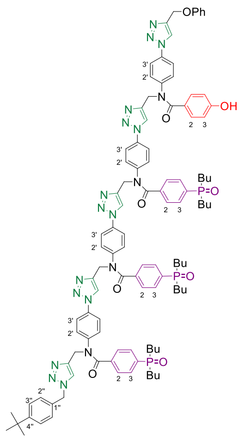

A solution of **11** (4 mg, $1.1\cdot10^{-3}$ mmol) in THF (2 mL) and water (0.5 mL) was treated with LiOH (1M in water, 35 µL, 0.035 mmol). After 20 minutes of stirring at rt, the solution was acidified with 1M HCl aq. soln. to pH 2-3 and extracted with EtOAc (3x). The organic layer was washed with brine and dried over $MgSO_4$. The residue was purified by flash column chromatography on silica gel (gradient from 0% to 15% of MeOH in CH_2Cl_2) to afford **12** (1 mg, 35%) as a white amorphous solid.

^{1}H NMR (500 MHz, $CDCl_3$): δ_H = 9.68 (s, 1H, OH), 8.20 (s, 1H, CH_{triaz}), 8.10 (s, 1H, CH_{triaz}), 8.00 (s, 2H, CH_{triaz}), 7.66 (s, 1H, CH_{triaz}, ^{t}Bu cap), 7.59 (m, 8H, 3’-H), 7.49 (m, 8H, 2-H and 3-H, PO), 7.38 (m, 4H, 2-H, PO), 7.38 (d, 2H, *J* = 8.5 Hz, 3’’-H ^{t}Bu cap), 7.30 (m, 8H, 2’-H; 3’’-H, PhO cap), 7.19 (d, 4H, *J* = 8.5 Hz, 2’-H; 2’’-H, ^{t}Bu cap), 7.13 (d, 2H, *J* = 8.5 Hz, 2-H, phenol), 7.00 (d, 2H, *J* = 8.5 Hz, 2’’-H, PhO cap), 6.97 (t, 1H, *J* = 8.5 Hz, 4’’-H, PhO cap), 6.65 (d, 2H, *J* = 8.5 Hz, 3-H, phenol), 5.48 (s, 2H, N-CH_2, ^{t}Bu cap), 5.31 (s, 2H, N-CH_2, PO), 5.27 (s, 2H, O-CH_2, PhO cap), 5.20 (s, 2H, N-CH_2) 5.19 (s, 2H, N-CH_2), 5.13 (s, 2H, N-CH_2, PO), 1.88 (m, 6H, 1’’-H, Bu), 1.74 (m, 6H, 1’’-H, Bu; overlapped by water peak), 1.45 (m, 6H, 2’’-H, Bu), 1.30 (s, 9H, ^{t}Bu), 1.25 (m, 18H, 2’’-H and 3’’-H, Bu;), 0.76 (m, 18H, 4’’-H, Bu).

^{13}C NMR (125.7 MHz, $CDCl_3$): δ_C = 170.4 (CO, phenol), 169.8, 169.4 and 169.3 (CO, PO), 160.1 (4-C, phenol), 158.3 (1’’-C, PhO cap), 152.2 (4’’-C, ^{t}Bu cap), 145.4, 145.3 and 145.3 (C_{triaz}), 144.4, 144.1 ($C_{triaz,}$; 1’-C), 143.8 and 143.7 (C_{triaz}, ^{t}Bu cap; 1’-C), 143.7 and 143.7 (1’-C), 138.4 (d, *J* = 2.5 Hz, 1-C, PO), 138.1 (d, *J* = 2.5 Hz, 1-C, PO), 138.0 (d, *J* = 2.5 Hz, 1-C, PO), 134.6, 135.5 and 135.3 (4’-C), 135.3 (d, *J* = 89.0 Hz, 4-C, PO), 134.7 (4’-C), 134.7 and 134.7 (d, *J* = 90.0 Hz, 4-C, PO), 131.5 (1’’-C, ^{t}Bu cap), 130.4, 130.3 and 130.3 (d, *J* = 9.0 Hz, 3-C, PO), 129.7 (3’’-C, PhO cap), 129.2, 129.1, 129.1, 129.0, 128.9, 128.8, 128.10 and 128.0 (C_{arom}), 126.2 (3’’-C, ^{t}Bu cap), 125.3 (1-C, phenol), 123.9 (CH_{triaz}, ^{t}Bu cap), 121.9 and 121.9 (CH_{triaz}), 121.5 (4’’-C, PhO cap), 121.4, 121.3, 121.2 and 121.2 (3’-C), 121.1 and 121.0 (CH_{triaz}, PO), 115.3 (3-C, phenol), 114.9 (2’’-H, PhO cap), 62.0 (O-CH_2, PhO cap), 54.1 (N-CH_2, ^{t}Bu cap), 46.7, 46.7, 465 and 46.4 (N-CH_2), 34.8 (C, ^{t}Bu), 31.4 (CH_3, ^{t}Bu), 29.4 (d, *J* = 68.5 Hz, 1’’-C, Bu), 29.4 (d, *J* = 68.5 Hz, 1’’-C, Bu), 29.3 (d, *J* = 68.5 Hz, 1’’-C, Bu), 24.1 (d, *J* = 14.5 Hz, 2’’-C, Bu), 24.0 (d, *J* = 14.5 Hz, 2’’-C, Bu), 23.6 (d, *J* = 4.0 Hz, 3’’-C, Bu), 23.5 (d, *J* = 4.0 Hz, 3’’-C, Bu), 13.7 and 13.7 (4’’-C, Bu).

^{31}P NMR (161.9 MHz, $CDCl_3$): δ_P = 41.8, 41.7 and 40.9.

HRMS (ES+): calcd $C_{108}H_{122}O_9N_{19}P_3$ 1945.8856 $[M+Na]^+$, found 1945.8841 $[M+Na]^+$.

FT-IR (ATR): ν_{max} 2957, 2924, 2879, 2855, 1645, 1518, 1283, 1240, 1150, 1106 and 1045 cm^{-1}.

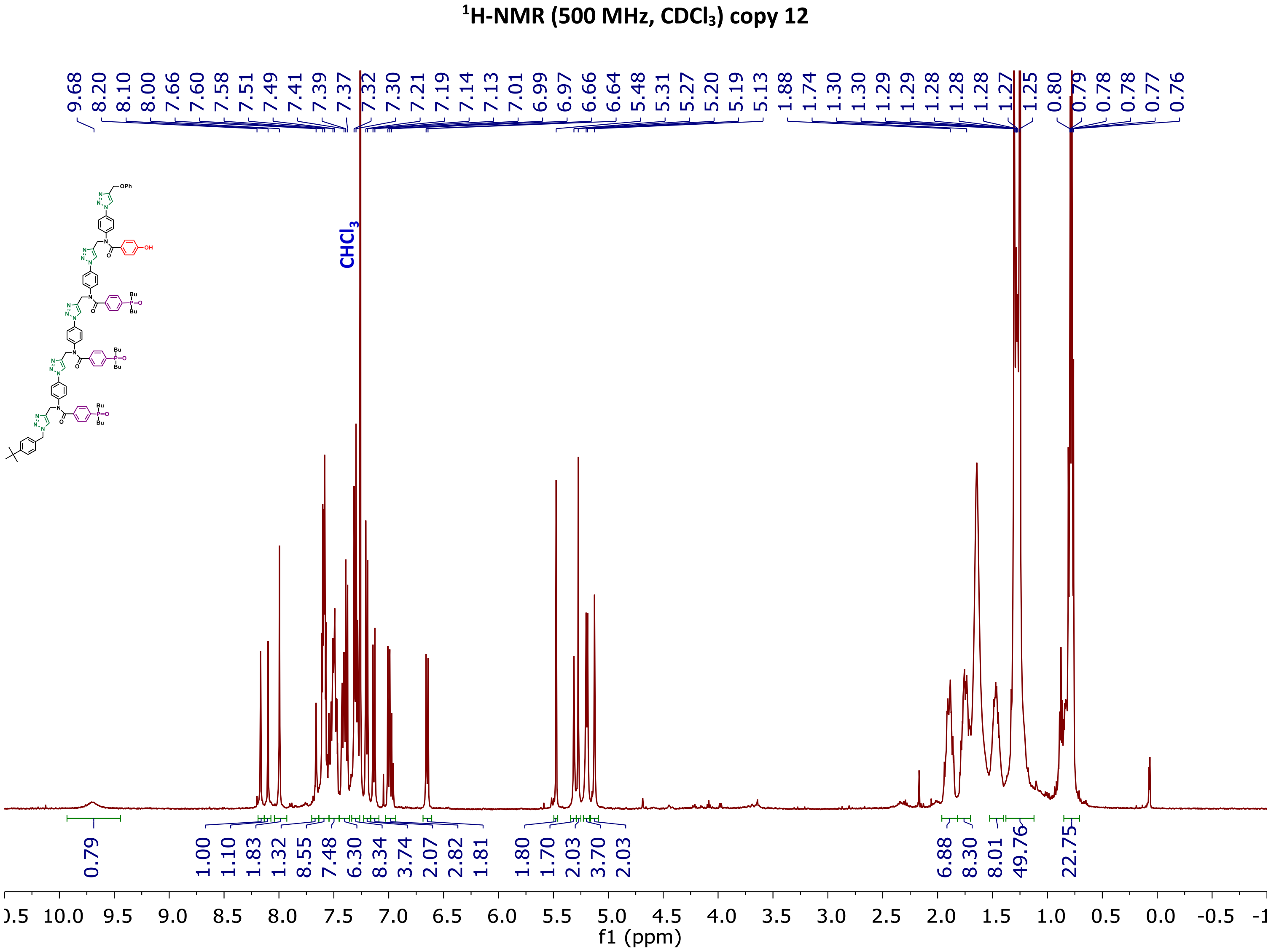

1H-NMR (500 MHz, CDCl3) copy 12
9.68
8.20
8.10
8.00
7.66
7.60
7.58
7.51
7.49
7.41
7.39
7.37
7.32
7.30
7.21
7.19
7.14
7.13
7.01
6.99
6.97
6.66
6.64
5.48
5.31
5.27
5.20
5.19
5.13
1.88
1.74
1.30
1.30
1.29
1.29
1.28
1.28
1.28
1.27
1.25
0.80
0.79
0.78
0.78
0.77
0.76
CHCl3
OPh
OH
Bu
0.79
1.00
1.10
1.83
1.32
8.55
7.48
6.30
8.34
3.74
2.07
2.82
1.81
1.80
1.70
2.03
3.70
2.03
6.88
8.30
8.01
49.76
22.75
f1 (ppm)

^{13}C-NMR (125.7 MHz, $CDCl_3$) copy 12

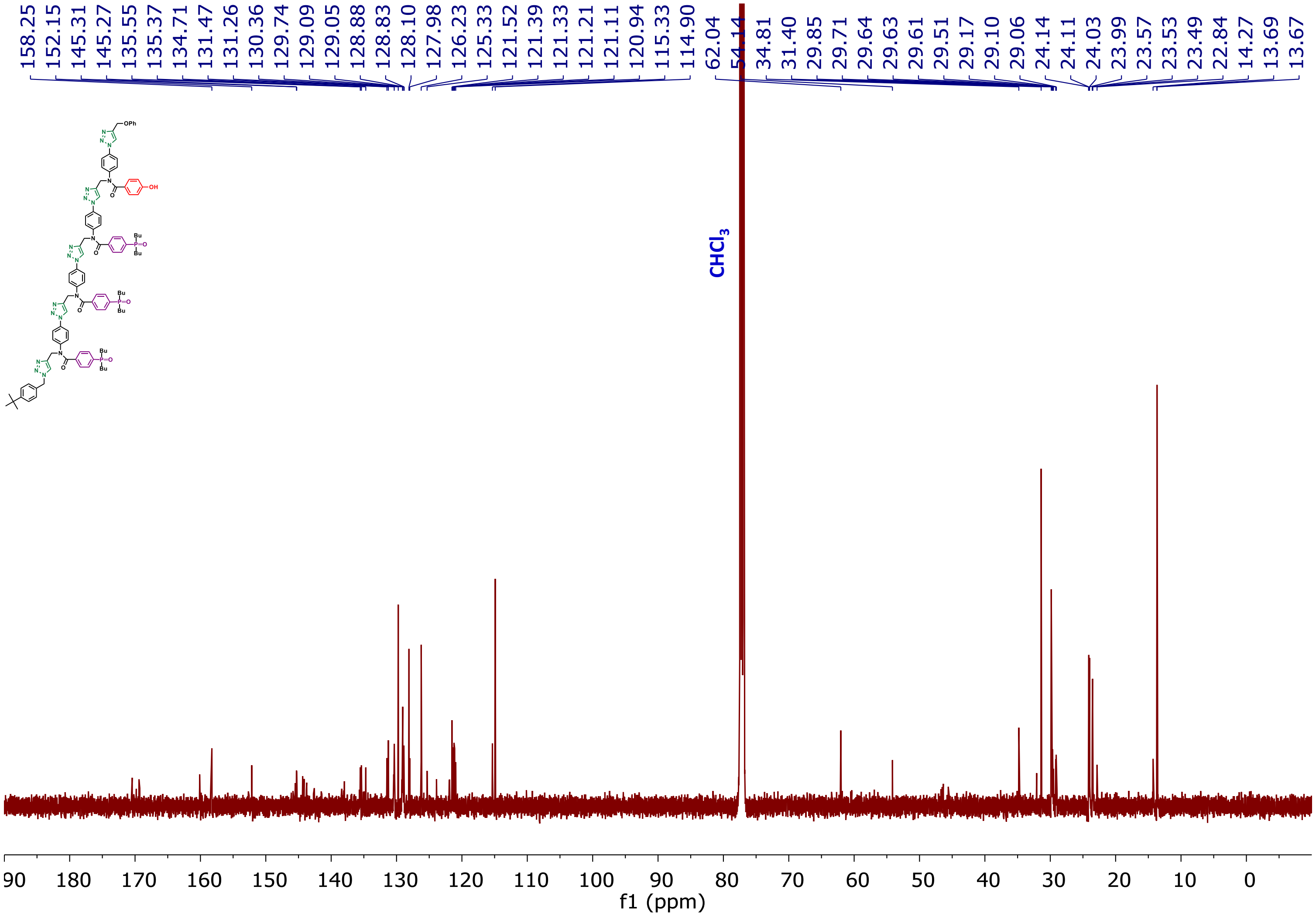

^{31}P NMR (161.9 MHz, $CDCl_3$) copy 12

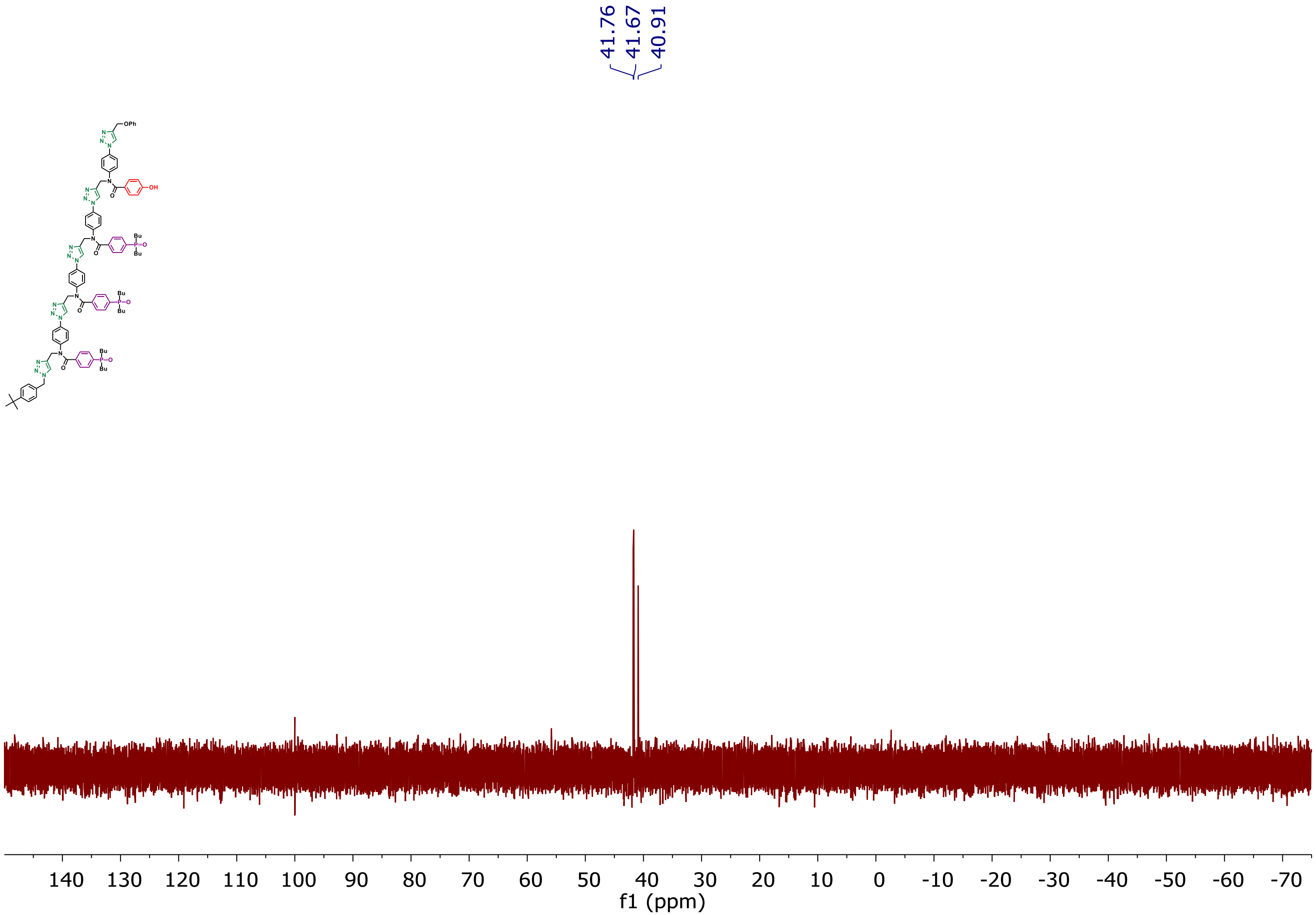

5. Full replication cycle from template 5

The full replication cycle from template 5 is illustrated in Scheme S5. The first step involves covalent attachment of the primer **6** by ester coupling to give the primed template **7**. The template-directed CuAAC reaction was then carried out using a mixture of the complementary phosphine oxide 3-mer **8** and a competing phenol 1-mer **9**. The final step is the cleavage of the covalent ester base-pair in the isolated duplex to release the template and the copy strand. The two oligomers were separated and the pure copy **12** was fully characterized. Fig 7 of the main text shows the relevant section of the UPLC traces for each of these steps. Fig S3 contains the full UPLC traces of the same steps.

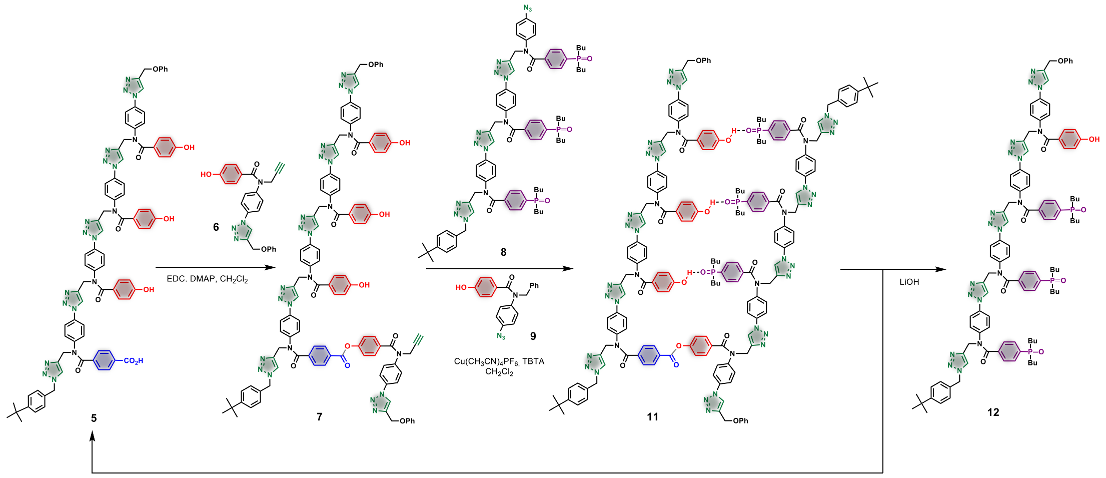

Scheme S5. Full replication cycle from template **5**.

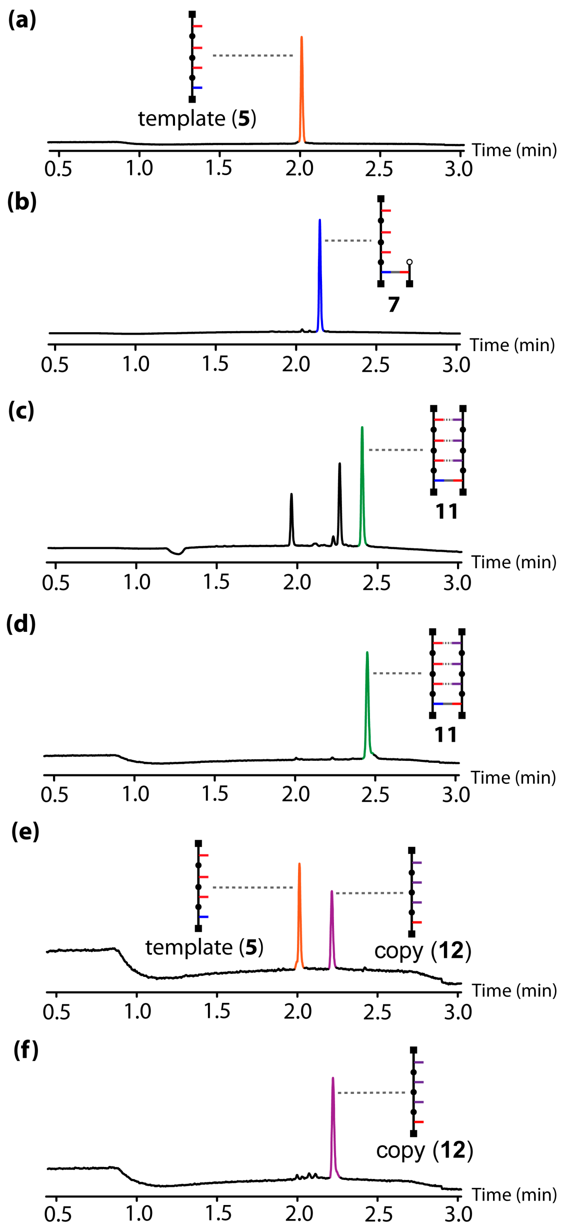

Fig S3. UPLC traces for H-bond template-directed oligomer synthesis using a covalent primer: (a) starting template **5**; (b) primed template **7**; (c) crude reaction mixture obtained after the CuAAC reaction of **7** in the presence of equimolar amounts of **8** and **9** (the additional peaks correspond to unreacted **8** and **9**); (d) isolated duplex **11** (e) crude reaction mixture obtained after hydrolysis of the ester base-pair; (f) isolated copy **12**. *UPLC conditions:* C18 column at 40 °C (254 nm) using water + 0.1% formic acid (A) and CH3CN + 0.1% formic acid (B); Gradient of 0–2 min 5% –100% B + 1 min 100% B.

6. Molecular modelling

Molecular mechanics calculations were performed using MacroModel version 13.1.141, (Release 2022-1, Schrödinger Inc.).[S3] All structures were minimized first and the minimized structures were then used as the starting molecular structures for all MacroModel conformational searches. The force field used was MMFFs as implemented in this software ($CHCl_3$ solvation). The charges were defined by the force field library and no cut off were used for non-covalent interaction. A Polak-Ribiere Conjugate Gradient (PRCG) was used, and each structure was subjected to 10000 iterations. The minima converged on a gradient with a threshold of 0.01. Conformational search was performed from previously minimized structures using 10000 steps. Images were created using PyMol.[S4]

Calculations were performed on systems with simplified capping groups, where the butyl chains on the phosphine oxides were replaced by methyl groups in order to reduce the computational cost. The calculation outcomes for each structure were sorted by energy and the 25 lowest-energy conformations were analysed.

7. References

[S1] Núñez-Villanueva, D.; Ciaccia, M.; Iadevaia, G.; Sanna, E.; Hunter, C. A. Sequence information transfer using covalent template-directed synthesis. *Chem. Sci*. **2019**, *10*, 5258.

[S2] Núñez-Villanueva, D.; Hunter, C. A. H-Bond templated oligomer synthesis using a covalent primer. *J. Am. Chem. Soc.* **2022**, *144*, 17307.

[S3] Schrödinger Release 2022-1: MacroModel, Schrödinger, LLC, New York, NY, 2022 (version 13.1.141).

[S4] The PyMOL Molecular Graphics System (open-source PyMOL). Version 2.3.0a0. Schrödinger, LLC.
